# T cell infiltration in both human multiple system atrophy and a novel mouse model of the disease

**DOI:** 10.1007/s00401-020-02126-w

**Published:** 2020-01-29

**Authors:** Gregory P. Williams, David J. Marmion, Aubrey M. Schonhoff, Asta Jurkuvenaite, Woong-Jai Won, David G. Standaert, Jeffrey H. Kordower, Ashley S. Harms

**Affiliations:** 1 Center for Neurodegeneration and Experimental Therapeutics, Department of Neurology, The University of Alabama at Birmingham (UAB), 1719 6th Ave. South, CIRC 446, Birmingham, AL 35294-0021 USA; 2 Department of Neurological Sciences, Rush University Medical Center, Chicago, IL 60612 USA

**Keywords:** Multiple system atrophy, T cells, Demyelination, Oligodendrocytes, Microglia, Monocytes, Alpha-synuclein

## Abstract

Multiple system atrophy (MSA) is a progressive neurodegenerative disorder characterized by abnormal accumulation of alpha-synuclein (α-syn) in oligodendrocytes accompanied by inflammation, demyelination, and subsequent synapse and neuronal loss. Little is known about the mechanisms of neurodegeneration in MSA. However, recent work has highlighted the important role of the immune system to the pathophysiology of other synuclein-related diseases such as Parkinson’s disease. In this study, we investigated postmortem brain tissue from MSA patients and control subjects for evidence of immune activation in the brain. We found a significant increase of HLA-DR^+^ microglia in the putamen and substantia nigra of MSA patient tissue compared to controls, as well as significant increases in CD3^+^, CD4^+^, and CD8^+^ T cells in these same brain regions. To model MSA in vivo, we utilized a viral vector that selectively overexpresses α-syn in oligodendrocytes (Olig001-SYN) with > 95% tropism in the dorsal striatum of mice, resulting in demyelination and neuroinflammation similar to that observed in human MSA. Oligodendrocyte transduction with this vector resulted in a robust inflammatory response, which included increased MHCII expression on central nervous system (CNS) resident microglia, and infiltration of pro-inflammatory monocytes into the CNS. We also observed robust infiltration of CD4 T cells into the CNS and antigen-experienced CD4 T cells in the draining cervical lymph nodes. Importantly, genetic deletion of TCR-β or CD4 T cells attenuated α-syn-induced inflammation and demyelination in vivo. These results suggest that T cell priming and infiltration into the CNS are key mechanisms of disease pathogenesis in MSA, and therapeutics targeting T cells may be disease modifying.

**Electronic supplementary material:**

The online version of this article (10.1007/s00401-020-02126-w) contains supplementary material, which is available to authorized users.

## Introduction

MSA is a rapidly progressive neurodegenerative movement disorder characterized by variable combinations of severe dysautonomia, parkinsonism, cerebellar ataxia, and pyramidal dysfunction [36, 61]. MSA is devastating, but rare, with an estimated mean incidence of 0.6–0.7 cases per 100,000 person-years [5]. Clinical and pathological features define two subtypes: MSA-C, a cerebellar variant characterized by olivopontocerebellar atrophy and prominent ataxia; and MSA-P, with parkinsonian symptoms and progressive striatal-nigral degeneration. These two phenotypes are not exclusive, as some cases can exhibit overlap of clinical and pathological features. Both subtypes involve severe demyelination and neurodegeneration, and there are no available therapies that slow or halt disease course. Death usually occurs between 7 and 9 years post-diagnosis [49].

MSA is part of the larger family of synucleinopathy disorders, which also includes Parkinson disease (PD) and Lewy Body Dementia (LBD). Each of these disorders is characterized by the accumulation and aggregation of misfolded forms of the protein α-syn. Unlike PD and LBD, misfolded α-syn is primarily found in oligodendroglia in MSA. These aggregates are referred to as glial cytoplasmic inclusions (GCIs) [3, 42, 57, 59].

Recent work has highlighted the important role of the immune system in the pathophysiology of synucleinopathies. This has been examined most closely in PD, where there is evidence for a synuclein-driven neuroinflammatory response [1, 7]. Lewy bodies and Lewy neurites, which are enriched with misfolded α-syn, are associated with HLA-DR^+^ microglia and the presence of CD4^+^ and CD8^+^ T lymphocytes surrounding neuromelanin^+^ neurons [6]. A recent study in PD patients revealed that T cell activation can be detected in circulating blood where CD4 and CD8 T cells are poised to respond to specific epitopes of α-syn and produce more cytokines (IFN-γ and IL-5) compared to healthy controls [55]. In PD animal models, targeting CD4 T cells has proven neuroprotective against inflammation and dopaminergic cell loss [6, 46, 48]. Studies in MSA are more limited, but there is evidence for increase in pro-inflammatory cytokines in the CSF [10, 65] and brain parenchyma [34, 47] of MSA patients as well as microgliosis and astrogliosis in postmortem MSA tissue [33, 47]. While evidence of inflammation has been observed in human disease and in transgenic animal models of MSA [52–54], it is still unknown what specific immune-mediated mechanisms are critical to disease pathogenesis.

In this current study, we examined innate and adaptive immune activation in postmortem tissue from MSA patients. In vivo, using a novel Olig001-SYN mouse model of MSA, we explored the mechanistic aspects of immune activation, which revealed a significant role for T cells similar to that seen in human MSA. More importantly, we found that this CD4-driven T cell response is required for α-syn-induced neuroinflammation and demyelination. These findings point to the potential of immune modulation as a disease-modifying treatment for MSA.

## Material and methods

### Human tissue processing and postmortem evaluation

Post-mortem brain tissue of MSA and control subjects were obtained from Rush University Medical Center and Banner Health. Autopsies of MSA subjects (*n* = 3) performed at Rush University Medical Centeras were performed as follows: the brains were removed from the calvarium and processed as described previously [9]. Briefly, each brain was cut into 1 cm coronal slabs and then hemisected. The slabs were fixed in 4% paraformaldehyde for 5 days at 4 °C. The brain slabs from one side were used for pathological diagnosis. The brain slabs from the other side were cryoprotected in 0.1 M phosphate‐buffered saline (PBS) pH 7.4 containing 2% dimethyl sulphoxide, 10% glycerol for 48 h followed by 2% dimethyl sulphoxide and 20% glycerol in PBS for at least 2 days before sectioning. The fixed slabs containing substantia nigra and striatum were cut into 18 adjacent series of 40-µm-thick sections on a freezing sliding microtome. All sections were collected and stored in a cryoprotectant solution before processing.

A complete neuropathologic evaluation was performed [28] confirming the presence of GCI as well as other neuropathology. These details can be found in Supplemental Table 1. Dissection of diagnostic blocks included a hemisection of brain, including the substantia nigra, striatum, cerebellar peduncle, and cerebellum. Glial cytoplasmic inclusions were examined with hematoxylin and eosin staining and further identified with antibodies to α-syn using alkaline phosphatase as the chromogen. A “definite” diagnosis of MSA was based on the presence of glial cytoplasmic inclusions, as well as a lack of Lewy bodies and Lewy neurites, and moderate or severe nigral neuronal loss, which corresponded with clinical diagnosis. Representative images confirming MSA glial cytoplasmic inclusion staining can be found in Supplementary Fig. 1a, online resource.

MSA (*n* = 3) and Control tissue (*n* = 6) from age-matched subjects were obtained from the Arizona Study of Aging and Neurodegenerative Disorders and Brain and Body Donation Program and collected as previously described [4]. All cases underwent a full neuropathological assessment at autopsy, evaluating relevant pathological proteins such as Tau, amyloid beta, and alpha-synuclein. “Neurological Control” is a subject without dementia or Parkinsonism during life and without a major neuropathological diagnosis. While pathology of tau or amyloid beta was present in some of the cases, it never reached a level sufficient to diagnosis any disease. Of note, our analysis (MSA cases and controls) were limited to one section per case due to the availability of tissue supplied from the brain bank, where only one level is sectioned per brain region.

A list of subject demographics is given in Supplemental Table 1.

### Immunohistochemistry of human samples

Free-floating striatal and nigral sections of control (*n* = 5) and MSA (*n* = 5) brain tissue were first rinsed of cryoprotectant solution and then underwent citric acid heat mediated antigen retrieval. Nonspecific background staining was blocked by a 1-h incubation in a solution containing 2% bovine serum albumin and 3% of either goat or horse serum. Tissue sections were incubated at room temperature overnight in the following primary antibodies: rabbit anti-Human CD3 (polyclonal, Dako A0452), mouse anti-CD4 (clone RIV6, Invitrogen MA1-7631), rabbit anti-CD8 (polyclonal, Abcam ab4055), and mouse anti-HLA-DR (clone LN3, Invitrogen MA5-11966). Sections were washed of primary antibody, then incubated with appropriate secondary antibodies (biotinylated goat anti-rabbit Vector Laboratories BA-1000; biotinylated horse anti-mouse Vector Laboratories BA-2000; for 1-h, washed again, and incubated with avidin–biotin complex (Vector Laboratories PK-6100) for 75-mins. The immunohistochemical reaction was completed with 0.05% 3,3′-diaminobenzidine (DAB) with 2% nickel enhancement and 0.005% H_2_O_2_. Sections were mounted on gelatin-coated slides, dehydrated through graded alcohol, cleared in xylene, and coverslipped with Cytoseal^™^ (Richard-Allan Scientific^™^). 40 μm sections of cynomolgus macaque spleen were used as positive controls for T cell staining (Supplementary Fig. 2, online resource).

### Immunofluorescence of human samples

Free-floating striatal and nigral sections of control (*n* = 3) and MSA (*n* = 3) brain tissue were first rinsed of cryoprotectant solution and then underwent citric acid heat mediated antigen retrieval. Nonspecific background staining was blocked by a 1-h incubation in a solution containing 2% bovine serum albumin and 3% of donkey serum. Tissue sections were incubated at room temperature overnight in the following primary antibodies: rabbit anti-Human CD3 (polyclonal, Dako A0452), mouse anti-HLA-DR (clone LN3, Invitrogen MA5-11966), rabbit anti-alpha-synuclein (phosphoS129) [EP1536Y] (polyclonal, abcam ab51253), mouse anti-alpha-synuclein (phosphoS129) [P-syn/81A] (monoclonal, abcam ab184674), rabbit anti-DARPP-32 [EP720Y] (polyclonal, abcam ab40801). Sections were washed of primary antibody, then incubated with appropriate Alexa-conjugated secondary antibodies (Life Technologies) at room temperature for 1 h. Sections were mounted on gelatin-coated slides, dehydrated through graded alcohol, cleared in xylene, and coverslipped with DPX mounting medium (Sigma-Aldrich). All images were obtained on a Nikon Eclipse Ti2 confocal microscope using a Nikon A1RHD camera. All figures were prepared using Photoshop 8.0 graphics software. Only minor adjustments of brightness and/or contrast were made.

### Stereology

To estimate the number CD3^+^, CD4^+^, and CD8^+^ T cells in control and MSA postmortem brain tissue in an unbiased fashion, stereological counting methods [63] using the optical fractionator probe in Stereo Investigator (Microbrightfield Bioscience, Version 10.40) were applied in a blinded fashion. The putamen or substantia nigra were outlined using a 1.25× objective and a random and systematic sampling of sections was employed where cells were counted from a random starting point at regular predetermined intervals (Putamen: *x* = 700 μm, *y* = 700 μm; Substantia Nigra: *x* = 400 μm, *y* = 400 μm) with a counting frame (80 μm × 80 μm) using a 60 × oil immersion objective.

### HLA-DR imaging and quantification

Free-floating striatal and nigral sections of control (*n* = 4) and MSA (*n* = 4) brain tissue were processed as noted above. The entire region of the putamen and substantia nigra were outlined and imaged using 4× objective on a Nikon eclipse Ti2 microscope using a Nikon D5-Ri2 color camera and NIS elements AR software version 5.10.01. The acquired image files were blinded and quantified by the mean gray value using imageJ (NIH) software. Briefly, the region of interest was outlined and converted to gray scale. The image was then inverted, vessels and holes in the tissue were excluded, and the background was subtracted using rolling ball radius of 25.

All images were obtained on a Nikon Eclipse Ti2 microscope using a Nikon D5-Ri2 color camera. All figures were prepared using Photoshop 8.0 graphics software. Only minor adjustments of brightness and/or contrast were made.

### Mice

Male and female C57BL/6 (#000664 Jackson Laboratories), *Ccr2* reporter knock in (B6.129(Cg)-Ccr2tm2.1Ifc/J, #017586 Jackson Laboratories) *Tcrb*^−/−^ (B6.129P2-Tcrbtm1Mom/J, #002118 Jackson Laboratories), and *Cd4*^−/−^ mice (B6.129S2-Cd4tm1Mak/J, #002663Jackson laboratories) were used for these studies and maintained on a congenic background. All researches conducted on animals were approved by the Institutional Animal Care and Use Committee at the University of Alabama at Birmingham.

### Olig001-vector

More detailed information of the development, construction, purification, and quality control of the Olig001 AAVs are described elsewhere [37, 43]. Briefly, the AAV-Olig001 vectors which have a high tropism for oligodendrocytes are packaged with a self-complementary genome with transgene expression mediated by the CBh promoter and bovine growth hormone polyA [24]. The AAV vectors were produced by the University of North Carolina Vector Core facility by triple-transfecting production plasmids (pXX6-80, pTRS-ks-CBh-EGFP, and AAV pXR-Olig001) into HEK293 cells. AAV vectors were then purified from the cells by iodixanol gradient centrifugation, followed by ion-exchange chromatography.

### Stereotaxic surgery

All surgical procedures on male and female C57BL/6 (WT), *Ccr2* reporter knock in, *Tcrb*^−/−^, or *Cd4*^−/−^ mice were performed similar to previously described work [25, 26, 64]. Briefly, mice were anesthetized with isoflurane and unilaterally (immunohistochemistry experiments) or bilaterally (flow cytometry experiments) injected with 2 μl of Olig001-GFP (1 × 10^13^ vg/ml) or Olig001-SYN (1 × 10^13^ vg/ml) into the dorsolateral striatum at a rate of 0.5 μl/min with a Hamilton syringe. The needle was left in the injection site for an additional 2 min and then slowly retracted over the course of 2 min. The stereotaxic coordinates used from bregma were AP + 0.2 mm, ML +/ −  2.0 mm, and DV −  2.7 mm from dura.

### Immunohistochemistry of mouse samples

At 4 weeks post viral transduction, mice were anesthetized, euthanized, and brains were collected for processing as previously described [25]. Briefly, animals were perfused with heparinized 0.01 M PBS, followed by 4% paraformaldehyde, drop-fixed overnight and transferred for cryoprotection to 30% sucrose in PBS pH 7.4. Brains were frozen on dry ice and coronal sections 40-μm thick were serially collected using a sliding microtome. Sections were stored in 50% glycerol in 0.01 M PBS at −  20 °C.

For fluorescent analyses, free-floating sections were washed with Tris-buffered saline (TBS) and labeled as previously published [25, 26, 64]. Briefly, sections were blocked in appropriate 5% normal serum and then labeled with anti-MHCII (clone M5/114.15.2, eBiosciences), anti-alpha-synuclein (phospho-Serine129 (pSer129), clone EP1536Y, Abcam), anti-tyrosine hydroxylase (TH, clone AB152, Sigma-Aldrich), anti-IBA1 (Wako), anti-TMEM119 (clone 28-3, Abcam), anti-CD3 (clone 17A2, eBioscience), anti-CD4 (clone RM4-5, BD Bioscience), anti-CD8 (clone 4SM15, eBioscience), or anti-MBP (clone SMI 99, BioLegend) antibodies diluted in 1% normal serum in TBS-Triton (TBST) overnight at 4 °C. Appropriate Alexa-conjugated secondary antibodies (Life Technologies) or LI-COR infrared secondary antibodies (LI-COR Biosciences) were applied at room temperature for 2.5 h. Sections were mounted onto coated glass slides, and cover slips were added using hard set mounting medium (Vector Laboratories).

For diaminobenzidine (DAB) staining, anti-MHCII (clone M5/114.15.2; eBiosciences) and anti-alpha-synuclein (pSer129, clone EP1536Y, Abcam) antibodies were diluted in 1% normal serum in TBST and incubated with sections overnight at 4 °C. Appropriate biotinylated secondary antibody (Vector Laboratories) were applied at room temperature for 2-h. Then, R.T.U. Vectastain ABC reagent (Vector Laboratories) and DAB kit (Vector Laboratories) were used according to the manufacturer's instructions to develop HRP reactions. Sections were mounted onto coated glass slides, dehydrated, and coverslipped using Permount mounting medium (Fisher).

For myelin staining, sections were stained and processed according to the Millipore Black Gold II Myelin Staining Kit per the manufacturer’s instructions.

### Confocal imaging

Confocal images were acquired using a Leica TCS-SP5 laser scanning confocal microscope. Images were saved using the Leica LASAF software, exported and processed using Adobe Photoshop/Illustrator.

### MHCII, myelin, TH, and pSer129 imaging and quantification (mice)

Images were acquired using a Nikon Eclipse Ti-E. For myelin, MHCII (I-A/I-E), and pSer129 staining quantification, slides were blinded, scanned, and quantified by the mean gray value method via imageJ (NIH) as previously described [64]. Three striatal sections per animal encompassing the dorsal striatum injection site and pSer129 expression were chosen for quantification. The myelin, MHCII, or pSer129 mean gray value (average pixel value in a selected region) was calculated for both ipsilateral and contralateral contours (drawn to match pSer129 expression) of the dorsal striatum and corpus callosum (normalized to non-tissue slide background mean gray value). Myelin, MHCII, or pSer129 fold induction was determined by dividing ipsilateral mean gray values by contralateral mean gray values and an average fold induction was determined per animal, *n* = 3–6 animals were quantified per treatment group.

For TH densitometry, LI-COR labeled striatal sections were blinded and then scanned with the Odyssey CLx system (LI-COR Biosciences). Three striatal sections per animal encompassing the dorsal striatum injection site were selected in Image Studio (LI-COR Biosciences) and the TH signal was calculated for both ipsilateral and contralateral contours of the dorsal striatum. TH fold expression was determined by dividing ipsilateral contour signal by the contralateral signal and an average fold induction was determined per animal, *n* = 3 animals quantified for treatment group.

For pSer129 positive cell counting, a 35,000 area per point grid was overlaid on the three striatal sections. Five random grids were assigned per ipsilateral section and an individual blinded to the genotypes counted the number of pSer129 positive cell bodies per random grid. The average of the counts per random field of view (grid) was used for the final quantifications between genotypes.

### Mononuclear cell isolation and flow cytometry

Mononuclear cells were isolated 4 weeks post-transduction from striatum or lymph nodes (LNs) of mice bilaterally transduced with Olig001-SYN or Olig001-GFP control according to published methods [26, 45, 64]. Briefly, striatums were triturated and digested with 1 mg/mL Collagenase IV (Sigma) and 20 μg/mL DNAse I (Sigma) diluted in RPMI 1640 with 10% heat inactivated fetal bovine serum, 1% l-glutamine (Sigma), and 1% Penicillin–Streptomycin (Sigma). Mononuclear cells were separated out using a 30/70% percoll gradient (GE) as previously described [30]. For deep cervical lymph nodes, lymph nodes were isolated and subsequently disassociated through a 70 μm filter. For intracellular cytokine staining, isolated mononuclear cells were stimulated with PMA (50 ng/mL, Fisher BioReagents) and ionomycin (750 ng/mL, Millipore Sigma) in the presence of GolgiStop (1:1000, BD Biosciences) for 4 h at 37 °C/5% CO_2_. For all staining, isolated cells were blocked with anti-Fcy receptor (clone 2.4G2 BD Biosciences) and then surfaced stained accordingly with fluorescent-conjugated antibodies against CD45 (clone 30-F11, eBioscience), CD11b (clone M1/70, BioLegend), MHCII (clone M5/114.15.2, BioLegend), Ly6C (clone HK 1.4, BioLegend), TCR-ß (clone H57-597, BioLegend), CD4 (clone GK1.5, BioLegend), CD8a (clone 53-6.7, BioLegend), CD44 (clone IM7, BD Biosciences), or CD62L (clone MEL-14, eBioscience). A fixable viability dye was used to distinguish live cells from debris per manufacturer’s instructions (Fixable Near-IR LIVE/DEAD Stain Kit, Invitrogen). For intracellular transcription factor and cytokine staining, cells were further processed using the Foxp3/Transcription Factor Staining Kit (eBioscience) or the BD Cytofix/Cytoperm Staining Kit (BD Biosciences) respectively and then stained accordingly with fluorescent-conjugated antibodies against FOXP3 (clone FJK-16s, eBioscience), T-bet (clone 4B10, BioLegend), GATA3 (clone 16E10A23, BioLegend), RORγt (clone Q31-378, BD Biosciences), IFN-γ (clone XMG1.2, eBioscience), IL-4 (clone 11B11, BioLegend), IL-17a (clone eBio17B7, eBioscience), or IL-10 (clone JES5-16E3, BioLegend). Samples were analyzed by flow cytometry using an Attune Nxt flow cytometer (Thermo Fisher Scientific) and FlowJo software (Tree Star).

### Statistical analysis

All graphs and corresponding statistical tests were generated or performed using Prism software (GraphPad).

For all analysis of human data, 4–5 subjects were analyzed per group. An unpaired, nonparametric Mann–Whitney test was used to compare groups. Graphs display the mean ± SEM. *p < 0.05, **p < 0.01.

For DAB MHCII staining quantification, four animals were analyzed per group. A one-way ANOVA with Dunnett’s test was used to compare groups. Graphs displayed the mean ± SEM. ***p < 0.001. For myelin staining quantification, 3–6 mice were analyzed per group. An unpaired t-test or a one-way ANOVA with Dunnett’s test was used to compare groups accordingly. Graphs displayed the mean ± SEM. *p < 0.05. Flow cytometry experiments utilized three to four independent samples per group, with two ventral midbrains pooled per sample. Therefore, each experiment used a total of 12–16 mice. Data was analyzed using an unpaired t-test or one-way ANOVA with Dunnett’s test was used to compare groups accordingly. Graphs displayed the mean ± SEM. *p < 0.05, ** < 0.01, ***p < 0.005, ****p < 0.001.

## Results

### HLA-DR expression on microglia in postmortem MSA brain tissue

**Fig. 1:**
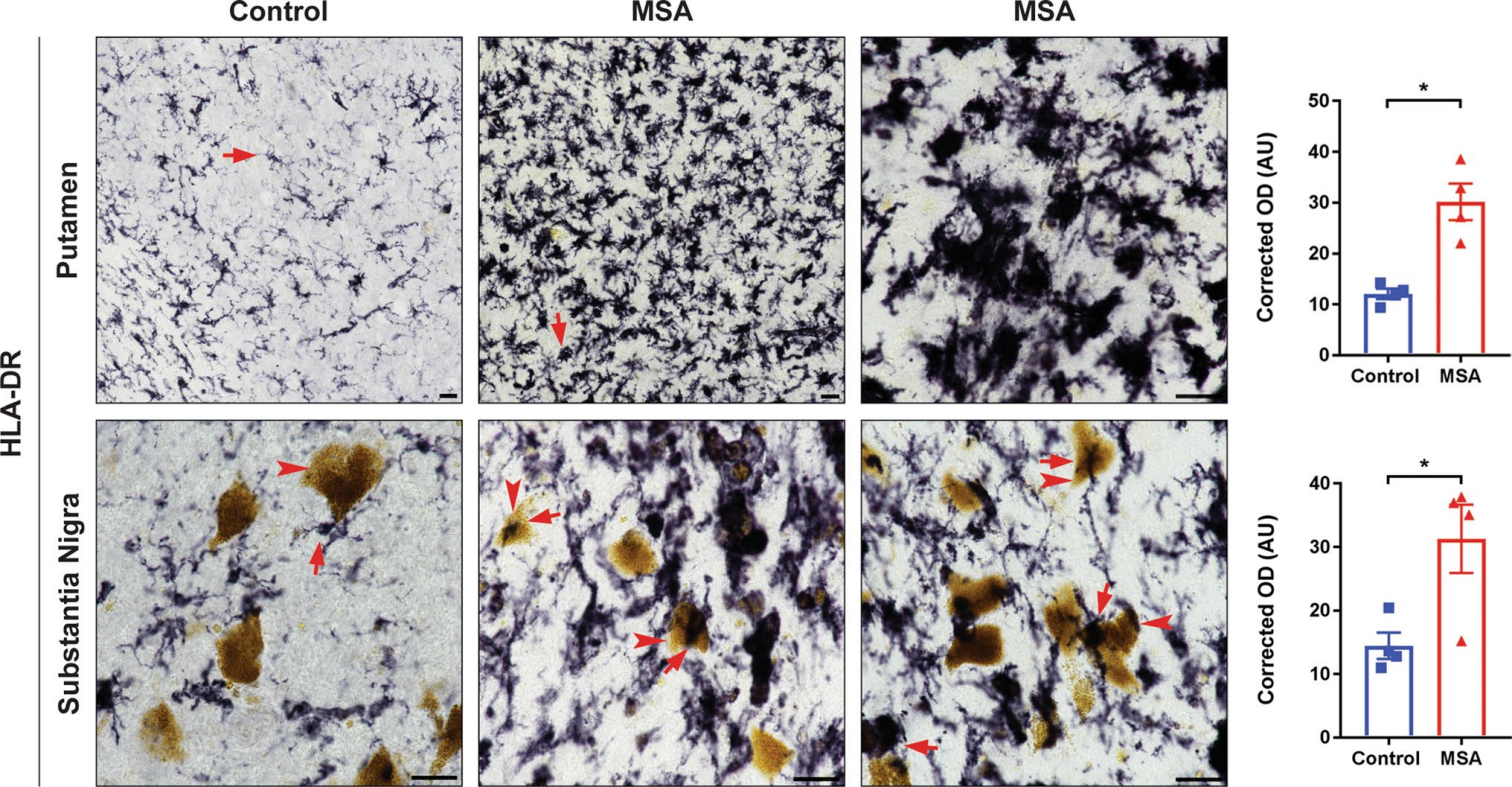
HLA-DR expression in human postmortem MSA brain tissue. Control (left) and MSA (middle and right) postmortem brain sections of the putamen (top) and substantia nigra (bottom) stained with HLA-DR (DAB, black staining). Quantification of the staining intensity revealed a significant increase of HLA-DR staining in the putamen and in the substantia nigra. Arrows denote “resting” (control, left) or “activated” (MSA, middle and right) microglia; arrowheads denote neuromelanin laden dopamine neurons in the substantia nigra. Representative images. All scale bars indicate 25 μm. Graphs display the mean ± SEM. *p < 0.05, unpaired nonparametric Mann Whitney Test was used to compare groups, *n* = 4 per group

As the immune response associated with α-syn has been recognized as a potential key driver in the pathogenesis in PD and related synucleinopathies, we wanted to explore if this was the case for MSA as well. Therefore, we first stained putamen and nigral sections of control and MSA postmortem brain tissue with HLA-DR (LN3), which recognizes a MHC class II antigen that visualizes microglia in these brain regions. MSA subjects displayed robust HLA-DR^+^ staining throughout both brain regions (Fig. 1). Optical density of HLA-DR^+^ staining revealed a significant 2.5-fold increase in putamenal HLA-DR expression (Control 12.06 ± 1.021 MSA: 30.18 ± 3.578, *p* =  0.0143) and a significant 2.17-fold increase in nigral HLA-DR expression (Control: 14.45 ± 2.07, MSA: 31.29 ± 5.383, *p* =  0.0286) in MSA cases compared to control subjects. Immunofluorescent labeling revealed these HLA-DR^+^ positive microglia are in close association with pSer129^+^ GCIs as well as DARPP-32^+^ neurons in MSA putamenal sections (Supplementary Fig. 1b, c, online resource). Furthermore, MSA cases not only appeared to have a greater number of microglia, but also those microglia appeared to be in an “activated state”, due to the morphological appearance of the large bushy cell body and shortened processes compared to a “resting state” morphology seen in control subjects with smaller cell bodies and longer processes in the putamen (arrows, top panel) as well as the substantia nigra (arrows, bottom panel). High magnification images of MSA cases clearly highlight the bushy morphology characteristic of activated microglia (right column) and Supplementary Fig. 1d, online resource. Brown coloring in the nigral sections are neuromelanin laden dopamine neurons (denoted by arrowheads). While microglia in control subjects appear to be close to neuromelanin^+^ neurons (arrow and arrowheads bottom panel) they have a resting morphology, while in MSA cases the microglia appear to be “activated” and touching the neuromelanin^+^ neurons, with the neuromelanin seeming to be reduced and more diffuse compared to controls (bottom panel arrows and arrowheads). These results highlight increased microgliosis in key pathological regions in the MSA diseased brain compared to control subjects.

### T cell infiltration in postmortem MSA brain tissue

**Fig. 2:**
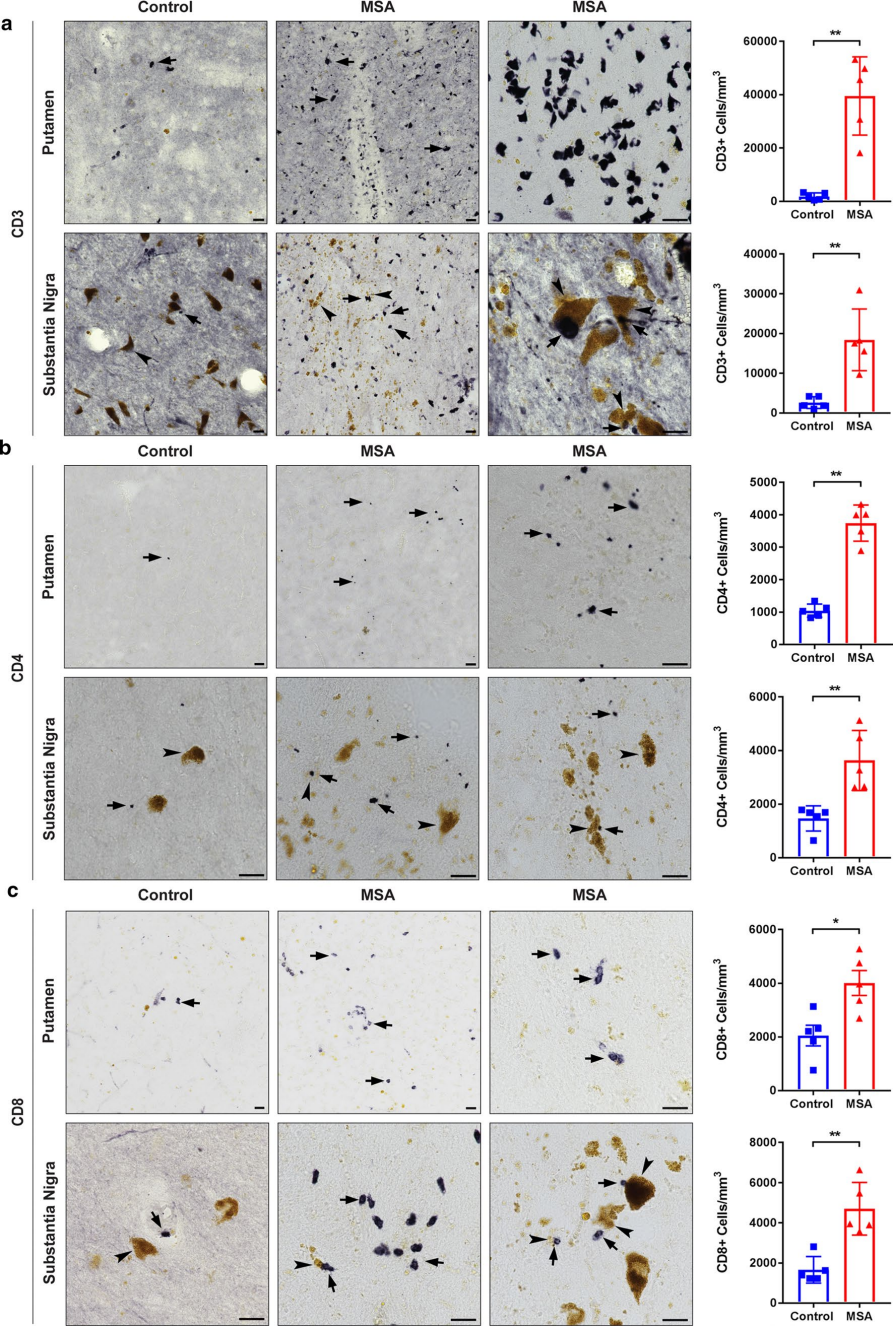
T cell infiltration in human postmortem MSA brain tissue. Control (left) and MSA (middle, right) postmortem brain sections of the putamen (top panel) and substantia nigra (bottom panel) were stained with CD3 (**a**), CD4 (**b**), and CD8 (**c**) antibodies (DAB, black staining). Unbiased stereological cell counts indicate a significant increase in the number of CD3^+^ T cells (**a**), CD4^+^ T (**b**), and CD8^+^ T cells (**c**) in the putamen and in the substantia nigra of MSA cases compared to control subjects. Brown coloring in the nigral sections are neuromalanin laden dopamine neurons (arrowheads). Of note, the yellow–brown pigment in putamenal sections is lipofuscin, an aging pigment composed of lipid residues of lysosomal digestion. Arrows denote T cells; arrowheads denote neuromelanin laden dopamine neurons in the substantia nigra. Representative images. All scale bars indicate 25 μm. Graphs display the mean ± SEM. *p < 0.05, **p < 0.01, unpaired nonparametric Mann Whitney Test was used to compare groups, *n* = 5 per group

The primary role of MHC proteins is presentation of antigen to patrolling T cells that possess the cognate T cell receptor (TCR) allowing recognition and a subsequent immune response to the source of antigen. Given that HLA-DR is an MHC class II cell surface receptor that presents antigens to CD4^+^ T cells, we sought to evaluate this T cell population in MSA brains. To do so, we first stained the MSA and control tissue with a human specific CD3 antibody. Robust CD3^+^ staining was seen throughout the entire putamen of MSA cases (Fig. 2a, top panel) as well as in close proximity to neuromelanin positive neurons in the substantia nigra (Fig. 2a, bottom panel). An occasional CD3^+^ T cell was seen near neuromelanin^+^ neurons in control subjects (arrow T cell, arrowhead neuromelanin, bottom left), but did not appear to be juxtaposed or directly on top of neuromelanin^+^ neurons as in MSA cases (arrows T cells, arrowheads neuromelanin, bottom right). In control cases, the neuromelanin appeared to be more densely packed and less diffuse when compared to MSA cases (Fig. 2a). CD3^+^ T cells were also located along blood vessels and deeper within the brain parenchyma in MSA tissue in contrast to control tissue where CD3^+^ T cells were mostly restricted to blood vessels (Supplementary Fig. 3a, online resource). Additionally, immunofluorescent labeling revealed that CD3^+^ T cells were in close association with pSer129^+^ GCIs in the putamen of MSA cases (Supplementary Fig. 3b, online resource). Unbiased stereological cell estimates on a single section per case revealed a statistically significant 21.36-fold increase in the number of CD3^+^ T cells/mm^3^ in the putamen of MSA cases when compared to control subjects (Control: 1850 ± 595.9, MSA: 39516 ± 6569, *p* = 0.0079) and a sevenfold increase in the substantia nigra (Control 2625 ± 656.8, MSA: 18404 ± 3474, *p* = 0.0079). While limited to striatal and nigral sections of MSA tissue, examination of nearby regions revealed the presence of CD3 + T cells in the caudate, internal capsule, internal and external segments of the globus pallidus, cerebellar peduncle, medial lemniscus, third cranial nerve rootlets, and the pons.

To characterize what specific subtype of T cells had infiltrated the brain in MSA, postmortem MSA and control tissue were stained for CD4 (Fig. 2b) and CD8 (Fig. 2c). Stereological estimates revealed a statistically significant ~ 3.58-fold increase of CD4^+^ T cells/mm^3^ in the putamen (Control: 1043 ± 90.48, MSA: 3743 ± 249.9, *p* = 0.0079) and a ~ 2.47-fold increase in the substantia nigra of MSA cases when compared to control cases (Control: 1469 ± 209.4, MSA: 2625 ± 500.5, *p* = 0.0079) (Fig. 2b). Similarly, there was a significant 1.95-fold increase of CD8^+^ T cells/mm^3^ in the putamen (Control 2055 ± 386.1, MSA: 4010 ± 464.1, *p* = 0.0159) and a 2.83-fold increase (Control: 1,660 ± 296.4; MSA: 4,702 ± 585.4, *p* = 0.0079) in the substantia nigra of MSA cases compared to controls (Fig. 2c). Brown coloring in the nigral sections are neuromalanin laden dopamine neurons (arrowheads). Of note, the yellow–brown pigment in putamenal sections is lipofuscin, an aging pigment composed of lipid residues of lysosomal digestion. As a control, all T cell antibodies were verified in positive control immunogenic tissue from cynomolgus macaque spleen before stainings were performed in the postmortem tissue and run in parallel with samples analyzed (Supplementary Fig. 2, online resource). Taken together, these results show an increased activation of microglia and MHC class II expression in MSA postmortem brain tissue with concomitant increases in CD4 and CD8 T cell populations.

### Olig001-SYN expression in oligodendrocytes results in demyelination in the striatum and corpus callosum of mice

**Fig. 3:**
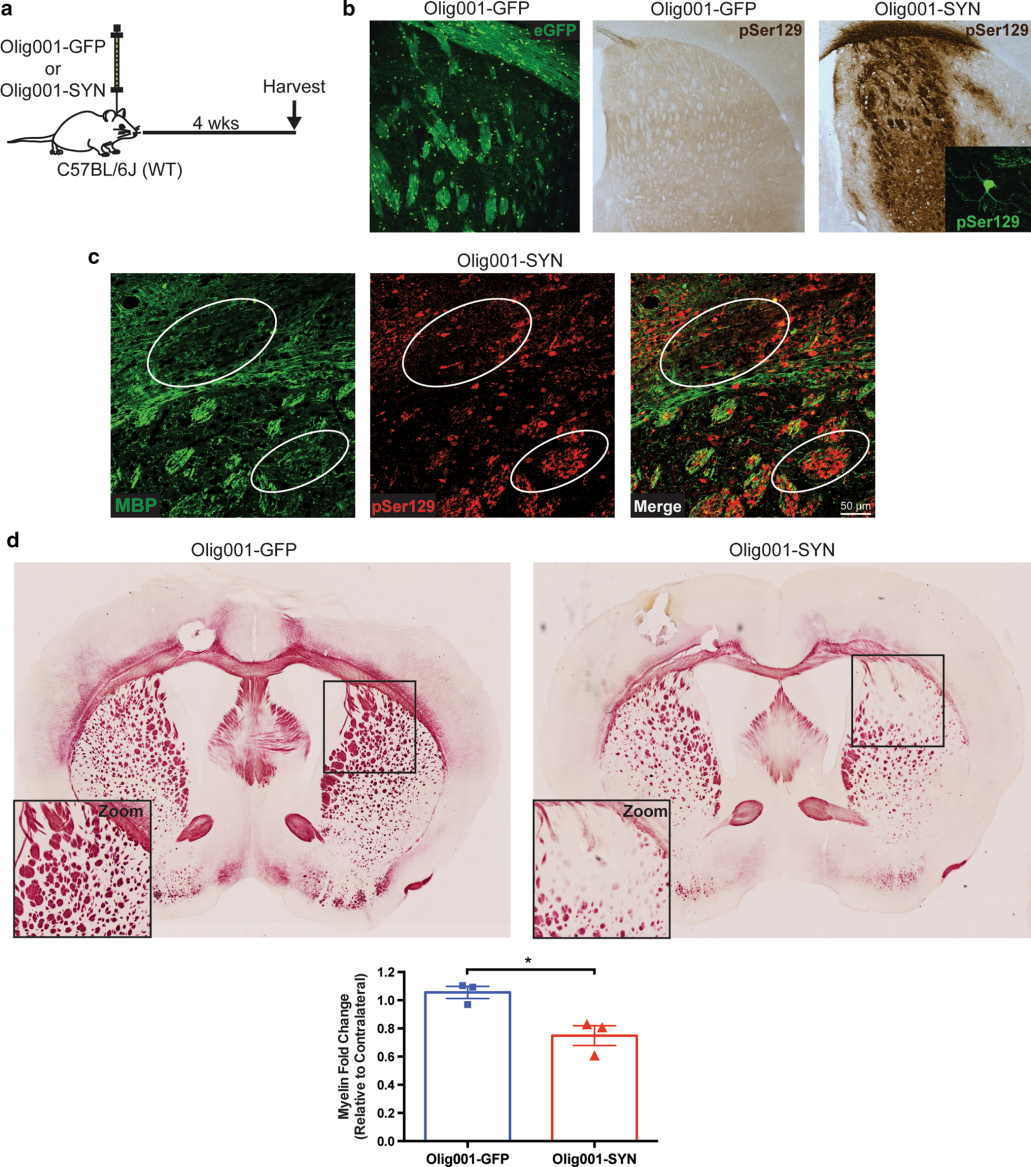
Olig001-SYN expression in oligodendrocytes results in demyelination in the striatum and corpus callosum of mice. **a** C57BL/6 J mice 8–12 weeks of age received a unilateral stereotaxic injection of Olig001-GFP (control) or Olig001-SYN into the right dorsal striatum. **b** 4 weeks post-transduction, eGFP (green) is visible in the dorsal striatum and corpus callosum of Olig001-GFP transduced animals and pSer129 (DAB, brown) in Olig001-SYN transduced animals, but not Olig001-GFP transduced animals. Zoom inset shows viral transduction in oligodendrocytes (pSer129, green). Representative images. **c** IHC shows areas with decreased MBP expression (white circle inserts) in the striatum and corpus callosum of mice transduced with Olig001-SYN. Olig001-SYN expression (pSer129, red) is associated with demyelination (MBP, green) 4 weeks post transduction. 40X confocal images. Scale bar is 50 μm **d** BlackGold^+^ staining in Olig001 transduced mice revealed areas of active demyelination (black square insert) in the corpus callosum and dorsal striatum. 10× brightfield images. BlackGold^+^ myelin is quantified and plotted as fold change relative to contralateral (uninjected side). Mean values ± SEM, unpaired *t* test, *p < 0.05, *n* = 3 per group

In order to determine whether oligodendrocyte expression of α-syn resulted in demyelination of the striatum and corpus callosum, we injected 2 μL of either Olig001-SYN or Olig001-GFP (control) into the right dorsal striatum of C57BL/6J (WT) mice modeling MSA-P, and performed immunohistochemistry (IHC) analyses 4 weeks post transduction (Fig. 3a). Similar to results obtained in rats and non-human primates [37], mice transduced with Olig001-SYN displayed robust positive staining for pSer129, an α-syn residue modification selectively and extensively observed in neuronal or glial synucleinopathies [2, 21], while control Olig001-GFP injected mice did not (Fig. 3b). Additionally, we observed the expression of myelin basic protein (MBP) co-localizing with pSer129 in the dorsal striatum and corpus callosum of Olig001-SYN transduced mice (Fig. 3c). Interestingly, it appeared that robust pSer129 expression in the dorsal striatum of Olig001-SYN transduced mice appeared in areas of lower MPB expression (circle inserts) indicating sites of active demyelination. This demyelination in the dorsal striatum was confirmed by performing Black Gold staining on both Olig001-GFP and Olig001-SYN injected animals (Fig. 3d). Consistent with the observed MBP results, Olig001-SYN transduced mice displayed a significant 1.4-fold reduction in myelin staining in the corpus callosum and dorsal striatum compared to the Olig001-GFP controls (GFP: 1.05 ± 0.04, SYN: 0.74 ± 0.07, *p* = 0.02). To assay for synaptic degeneration prior to neuronal loss, we performed tyrosine hydroxylase (TH) IHC on striatal sections from Olig001-GFP and Olig001-SYN 4 weeks post-transduction. Unbiased densitometry in the dorsal striatum revealed no difference in TH expression in the ipsilateral striatum surrounding pSer129 + inclusions (Supplementary Fig. 4, online resource). These results confirm that α-syn expression in oligodendrocytes via Olig001-SYN transduction in the dorsal striatum results in the expression of abnormal α-syn, as well as demyelination in the white matter tracts of the dorsal striatum and corpus callosum in mice.

### CNS myeloid activation is associated with oligodendrocyte expression of α-syn

**Fig. 4:**
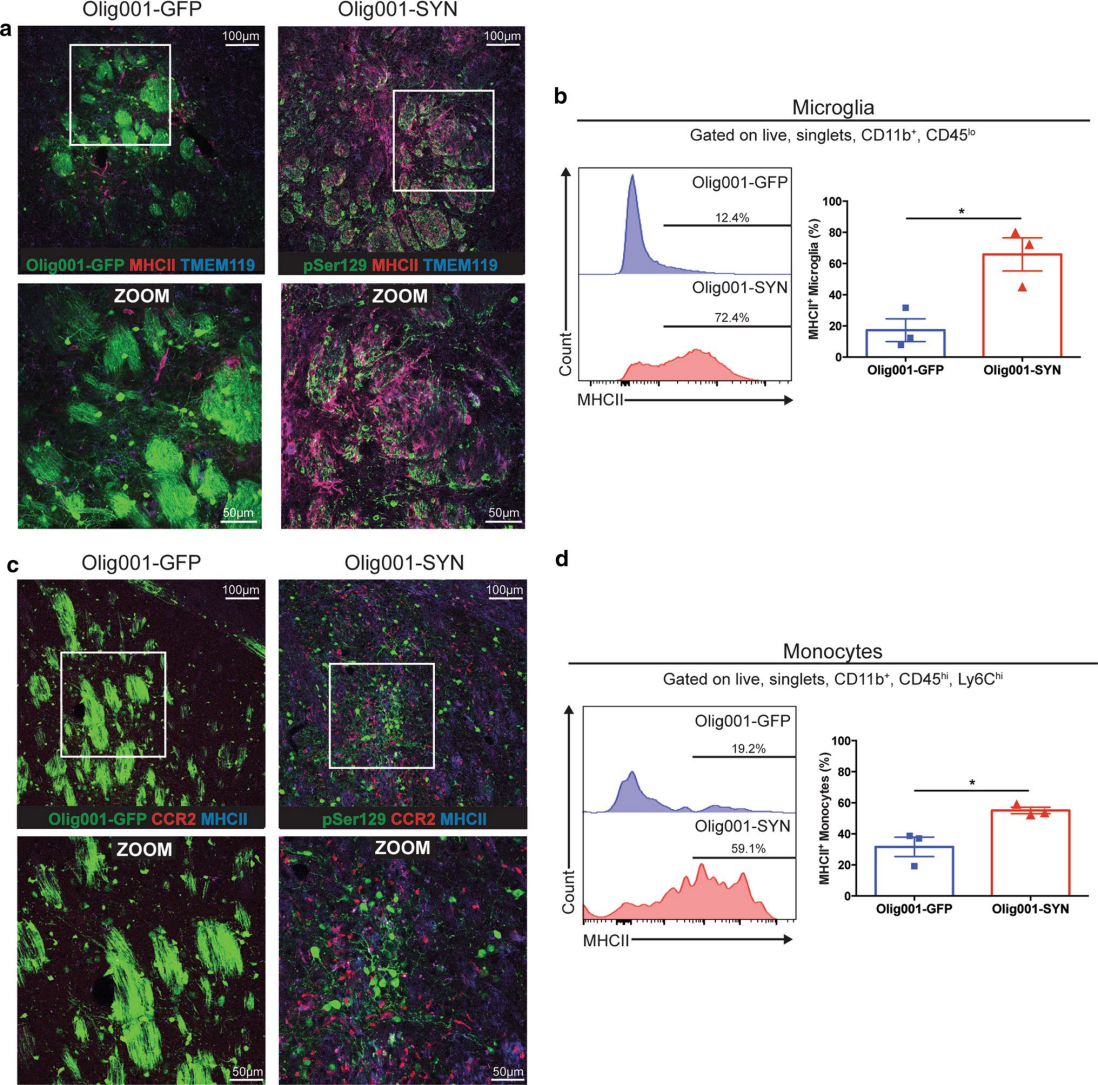
Olig001-SYN expression in the striatum induces MHCII expression on CNS resident microglia and infiltrating monocytes. C57BL/6J mice 8–12 weeks of age received a unilateral stereotaxic injection of Olig001-GFP (control) or Olig001-SYN into the right dorsal striatum. 4 weeks post-transduction **a** MHCII^+^ (red), TMEM119^+^ (blue) microglia are observed in dorsal striatum and corpus callosum of Olig001-SYN (pSer129, green) transduced animals. Zoom inserts (white boxes) show cells with distinct microglial morphology surrounding eGFP^+^ or pSer129 oligodendrocytes. Confocal images displaying co-localization of TMEM119^+^ microglia with MHCII. Representative images, Scale bar is 100 μM in 20× confocal images and 50μM in zoom inserts. **b** Mononuclear isolation and flow cytometry on isolated dorsal striatal tissues revealed a significant number and percentage of single, live, CD45^lo^, CD11b^+^, MHCII^+^ microglia in Olig001-SYN transduced mice when compared to Olig001-GFP control. Mean values are plotted ± SEM, unpaired *t* test. *p < 0.05, *n* = 3 per group. **c** C57BL6/J mice 8–12 weeks of age received a unilateral stereotaxic injection of Olig001-GFP (control) or Olig001-SYN into the right dorsal striatum. 4 weeks post-transduction CCR2^+^ (red), MHCII^+^ (blue) monocytes are observed in dorsal striatum and corpus callosum of Olig001-SYN (pSer129, green) transduced animals. Confocal images displaying co-localization of CCR2^+^ monocytes with MHCII. Representative images, Scale bar is 100 μM in 20× confocal images and 50 μM in zoom inserts. **d** Mononuclear cell isolation and flow cytometry revealed a significant increase in the percent of single, live, CD45^hi^, CD11b^+^, Ly6C^hi^, MHCII^+^ monocytes. Mean values are plotted ± SEM, unpaired t-test *p < 0.05, *n* = 3 per group

Based on our findings of activated HLA-DR positive microglia in human MSA postmortem tissue, we next wanted to explore whether there was a similar immune response to oligodendrocyte-mediated expression of α-syn in the dorsal striatum of mice. Similar to Fig. 3, we performed IHC on 40 μm striatal tissue sections 4 weeks post Olig001 transduction. We observed that in areas of pSer129 expression including the, there was marked MHCII expression on TMEM119^+^, CNS resident microglia (Fig. 4a)—indicative of an activated, inflammatory phenotype [39]. This robust MHCII expression was not observed in Olig001-GFP control tissue, where those microglia displayed low levels of MHCII in the CNS. MHCII expression on microglia could also be observed in the corpus callosum of Olig001-SYN injected animals (Supplementary Fig.5a, online resource). The increase of activated MHCII^+^ microglia in Olig001-SYN treated mice was confirmed by performing mononuclear cell isolation and flow cytometry on tissue isolated from the dorsal striatum and corpus callosum 4 weeks post Olig001-SYN transduction (Fig. 4b). In response to α-syn expression in oligodendrocytes, microglia, gated as CD45^lo^, CD11b^+^ (Supplementary Fig. 6, online resource for gating strategy used for FACS analysis), revealed a significant ~ fourfold increase in the MHCII expression when compared to GFP expressing controls (GFP: 17.2 ± 7.3, SYN: 65.8 ± 10.6, *p* =  0.02). To further characterize the myeloid response, we next wanted to see if monocytes responded to this inflamed CNS area. Using *Ccr2*^RFP^ reporter mice, we detected the infiltration of CCR2^+^ monocytes surrounding pSer129^+^ oligodendrocytes in the dorsal striatum in response to α-syn expression, but not Olig001-GFP animals (Fig. 4c). Additionally, mononuclear cell isolation and flow cytometric analysis of tissue isolated from the dorsal striatum and corpus callosum 4 weeks post injection revealed that CD45^hi^, CD11b^+^, Ly6C^hi^ monocytes had a significant 1.7-fold increased expression of MHCII in Olig001-SYN injected animals compared to Olig001-GFP controls (GFP: 31.6 ± 6.2, SYN: 55.1 ± 2.1, *p* = 0.02) (Fig. 4d). In terms of changes in total numbers of myeloid cells in the CNS in response to Olig001-SYN, we observed no increase in the total number of microglia (GFP: 20877 ± 4040, SYN: 22975 ± 6802, *p* = 0.8) (Supplementary Fig. 7, online resource). However, we did observe an increase in the total number of infiltrating monocytes and macrophages in Olig001-SYN transduced mice compared to Olig001-GFP control mice (GFP: 338.4 ± 18.1, SYN: 969.7 ± 152.2, *p* =  0.014) (Supplementary Fig. 7, online resource). Overall, these results suggest that both a local, CNS-resident myeloid response (microglia), as well as robust infiltration of peripheral myeloid cells (monocytes and macrophages) is occurring due to oligodendrocyte selective α-syn expression.

### T cells infiltrate into the CNS and accumulate in draining LNs in response to oligodendrocyte expression of α-syn

**Fig. 5:**
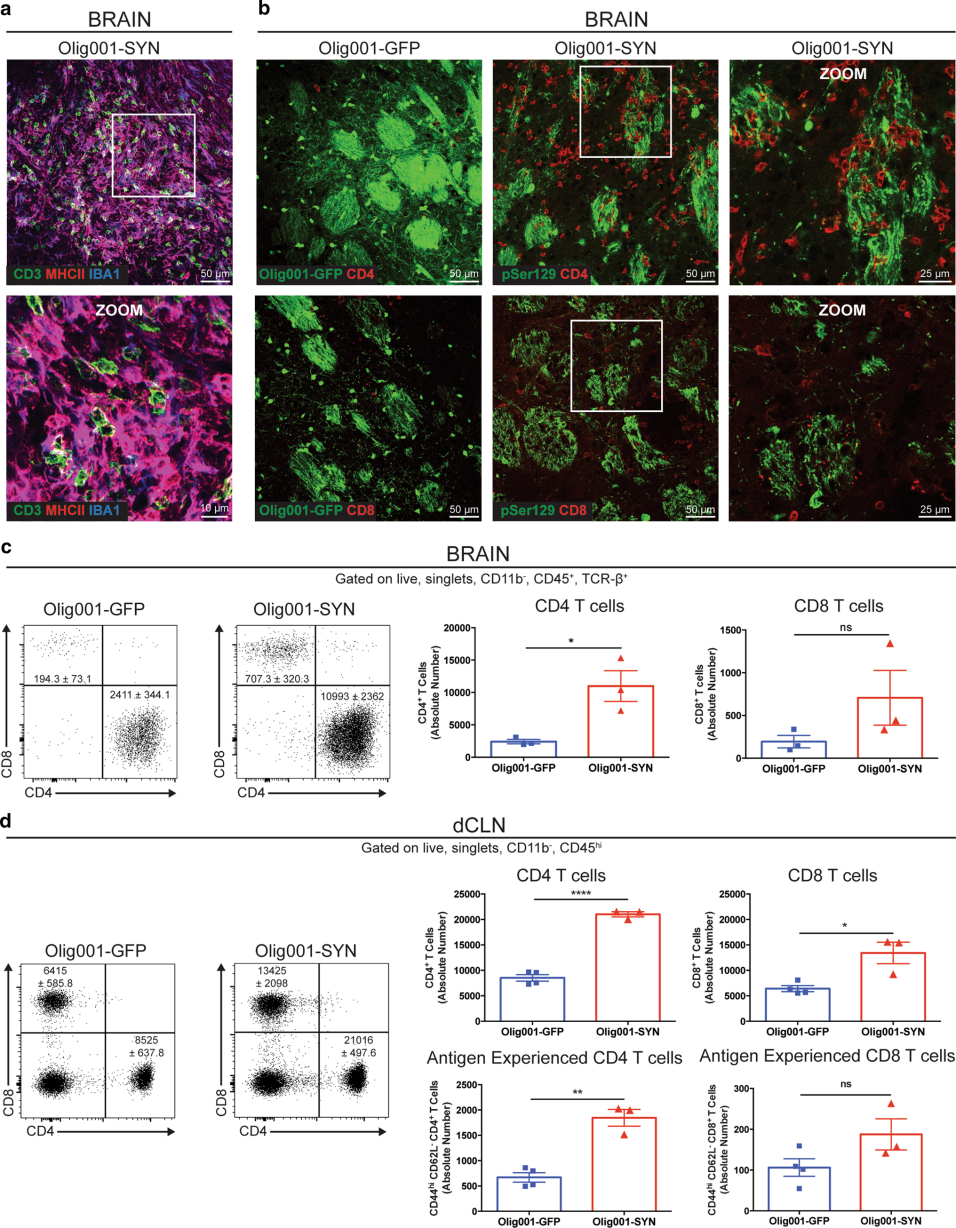
T cells infiltrate into the CNS and accumulate in draining LNs in response to oligodendrocyte expression of α-syn. **a** C57BL/6 J mice 8–12 weeks of age received a unilateral stereotaxic injection of Olig001-SYN into the right dorsal striatum. 4 weeks post-transduction in the CNS, CD3^+^ (green) T cells are observed in close proximity with IBA-1^+^ (blue) MHCII + (red) microglia/macrophages in the ipsilateral striatum. Bottom panel (white box, zoom insert) displays a cluster of CD3^+^ cells (green) in close proximity of IBA-1^+^ MHCII + microglia/macrophages in the dorsal striatum. 40 × confocal images. Scale bar is 50 μM in top paneel images and 10 μM in zoom insert. **b** Representative confocal images show CD8^+^ or CD4^+^ T cells (red) surrounding oligodendrocytes transduced with Olig001-SYN (pSer129, green) but not Olig001-GFP control. 40 × Confocal images. White box is zoom insert. Scale bar is 50 μM in 20 × images and 25 μM in zoom inserts. **c** C57BL/6 J mice 8–12 weeks of age received a bilateral stereotaxic injection of Olig001-SYN or Olig001-GFP control into the right dorsal striatum. 4 weeks post-transduction in the CNS, mononuclear cell isolation and flow cytometry on striatal tissues revealed an increase in the percent and number of single, live, CD45^+^, TCR-β^+^, CD4^+^ or CD8^+^ T cells in response to Olig001-SYN expression in the dorsal striatum. Mean values are plotted ± SEM, unpaired t-test. *p < 0.05. *ns* not significant, *n* = 3 per group. **d** C57BL/6 J mice 8–12 weeks of age received bilateral stereotaxic injections of Olig001-GFP (control) or Olig001-SYN into the right dorsal striatum. 4 weeks post-transduction draining cervical lymph nodes were isolated and analyzed by flow cytometry. Analysis revealed an increase in the absolute number of CD4^+^ T cells (gated as single, live, CD45^hi^, CD4^+^), as well as antigen-experienced CD4^+^ T cells (gated as single, live, CD45^+^, TCR-β^+^, CD4^+^, CD44^hi^, CD62L^−^). Mean values are plotted ± SEM, unpaired t-test. ****p < 0.0001, **p < 0.01. Analysis also revealed an increase in the absolute number of CD8^+^ T cells (gated as single, live, CD45^hi^, CD8^+^), but no increase in antigen experienced T cells (gated as single, live, CD45^+^, TCR-β^+^, CD4^+^, CD44^hi^, CD62L^−^). unpaired *t* test. *p < 0.05, *n* = 3–4 per group

Given our observation of T cells in postmortem MSA tissue, and our findings of increased HLA-DR or MHCII on myeloid cells in the CNS, we wanted to determine whether there was a reciprocal T cell response observed in our mouse model of MSA. IHC analysis of striatal tissue in Olig001-SYN injected mice 4 weeks post-transduction revealed numerous CD3^+^ T cells (Fig. 5a) in close proximity to IBA1^+^ CNS myeloid cells (zoom insert). More specifically, these Olig001-SYN responding CD3^+^ T cells were both CD4 and CD8 positive (Fig. 5b). Similarly, it is interesting to note the close proximity of CD4^+^ and CD8^+^ T cells to the pSer129^+^ oligodendrocytes, myelin fiber tracts, and the corpus callosum (Fig. 5b zoom insert, Supplementary Fig. 5b, online resource). In addition, to determine the ratio of CD4 to CD8 T cells, we performed mononuclear cell isolation and flow cytometric analysis on tissue isolated from the dorsal striatum and corpus callosum of Olig001-GFP and Olig001-SYN injected animals 4 weeks post-transduction. We observed a significant ~ 4.5-fold increase in CD4^+^ T cells in response to α-syn expression (GFP: 2411 ± 344, SYN: 10993 ± 2362, *p* = 0.02) (Fig. 5c). We observed a trend of increased CD8^+^ T cells responding to α-syn expression when compared to Olig001-GFP transduced controls, but statistical significance was not met (GFP: 194.3 ± 73.1, SYN: 707.3 ± 320.3, *p* = 0.2) (Fig. 5c). Along with number of CD4^+^ T cells in the dorsal striatum, we also observed a significant ~ 2.5-fold increase in the number of CD4^+^ T cells (GFP: 8525 ± 638, SYN: 21016 ± 498, *p* = < 0.0001) and a ~ twofold increase in the number of CD8^+^ T cells (GFP: 6415 ± 586, SYN: 13425 ± 2098, *p* ± 0.013) in the CNS draining deep cervical lymph nodes (dCLNs) of Olig001-SYN treated mice compared to Olig001-GFP controls (Fig. 5d). The increase in dCLN CD4 T cells partially consisted of an antigen-experienced phenotype (CD44^hi^, CD62L^−^) CD4 T cells (GFP: 669 ± 92.6, SYN: 1846 ± 165.5, *p* = 0.0011), but this was not the case for dCLN antigen-experienced CD8 T cells (GFP: 106.2 ± 21.4, SYN: 187.5 ± 38.1, *p* = 0.1) (Fig. 5d), indicating that Olig001-SYN responding dCLN CD4^+^ T cells have encountered antigen more so than dCLN CD8^+^ T cells. With this evidence, we show that oligodendroglia expression of α-syn results in not only a CNS myeloid response, but also a CD4 T cell response that can be observed in both the primary site of inflammation (CNS) as well as the main draining secondary lymphoid organ. These findings closely mirror our observed myeloid and T cell response in MSA postmortem tissue and support the concept that the immune system is playing a key role in the pathophysiology of MSA.

### α-syn responding CD4 T cells are associated with a Th1 phenotype

**Fig. 6:**
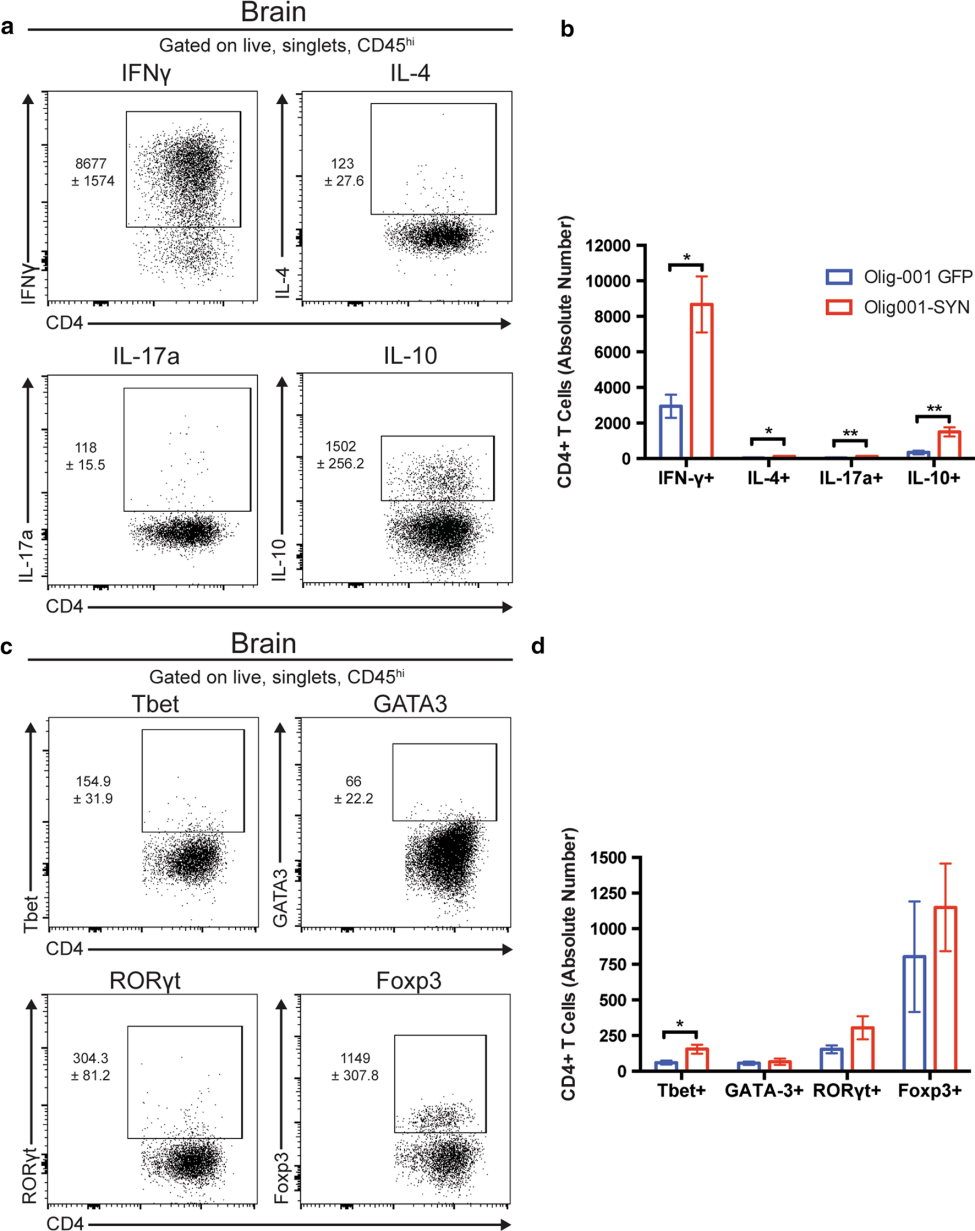
α-Syn responding CD4 T cells are associated with a Th1 phenotype. C57BL/6 J mice 8–12 weeks of age received a bilateral stereotaxic injection of Olig001-SYN or Olig001-GFP control into the right dorsal striatum. 4 weeks post-transduction in the CNS, mononuclear cell isolation, intracellular cytokine/transcription factor staining, and flow cytometry were performed on the isolated dorsal striatal tissues. **a** Representative flow cytometry plots depicting partial gating of the stained Th cytokines IFN-γ, IL-4, IL-17a, and IL-10. **b** Quantification of cytokine staining showing increased amounts of Th cytokines in the Olig001-SYN transduced animals compared to Olig001-GFP controls. Mean values are plotted ± SEM, unpaired *t* test. *p < 0.05, **p < 0.01, *n* = 4 per group. **c** Representative flow cytometry plots depicting partial gating of the stained transcription factors Tbet, GATA3, RORγt, and Foxp3. **d** Quantification of the transcription factor staining in **c** showing a significant increase in Th1 associated, Tbet^+^ CD4 T cells in the Olig001-SYN transduced animals compared to Olig001-GFP controls. Mean values are plotted ± SEM, unpaired t-test. *p < 0.05, *n* = 4 per group

T cell responses come in a variety of types primarily based on the pathogen and the tissue environment where the immune reaction is occurring. For example, Th1 CD4 T cells promote cell-mediated immunity and in autoantigenic cases have been heavily implicated in the inflammatory CNS disease multiple sclerosis [20]. In contrast, Th2 CD4 T cells are associated with promoting strong antibody responses and, in autoinflammatory cases, play a key role in the pathogenesis of asthma [18]. Given this important heterogeneity of T cell responses, we sought to better characterize the α-syn-mediated T cell response in our model of MSA. We performed intracellular cytokine and transcription factor staining on mononuclear cell isolates from striatal tissue 4 weeks post Olig001-GFP or Olig001-SYN transduced mice (Fig. 6). Cytokine analyses revealed a significant increase in all Th1/2/17/Treg associated cytokines IFN-γ, IL-4, IL-17a, and IL-10 respectively (Fig. 6a, b). However, the predominant cytokine response was in the IFN-γ^+^ CD4 T cell population with a threefold increase in Olig001-SYN injected animals compared to controls (GFP: 2944 ± 647.7, SYN: 8677 ± 1574, *p* = 0.015) (Fig. 6b). Interestingly, there was no significant difference in the amount of IFN-γ^+^ CD8 T cells in Olig001-SYN injected mice compared to GFP controls (Supplementary Fig. 8, online resource). This predominant CD4 IFN-γ response was also supported by tandem transcription factor staining on T cells isolated from striatal tissue 4 weeks post Olig001-GFP or Olig001-SYN transduced mice where were assayed for the Th1/2/17/Treg transcription factors Tbet, GATA-3, RORγt, and Foxp3 respectively (Fig. 6c, d). We observed a significant 2.5-fold increase only in the Th1 associated Tbet^+^ CD4 T cell population (Fig. 6d) in Olig001-SYN transduced mice compared to GFP controls (GFP: 60.3 ± 13.8, SYN: 154.9 ± 31.93, *p* = 0.03), consistent with our IFN-γ findings (Fig. 6b). These results indicate that the observed Olig001-SYN associated CD4 T cells possess a pro-inflammatory Th1 phenotype that may be driving and contributing to the Olig001-SYN associated myeloid response and subsequent degenerative processes observed in the model.

### Knockout of αβ T cells or CD4 T cells alone reduces CNS myeloid activation and demyelination in response α-syn expression

**Fig. 7:**
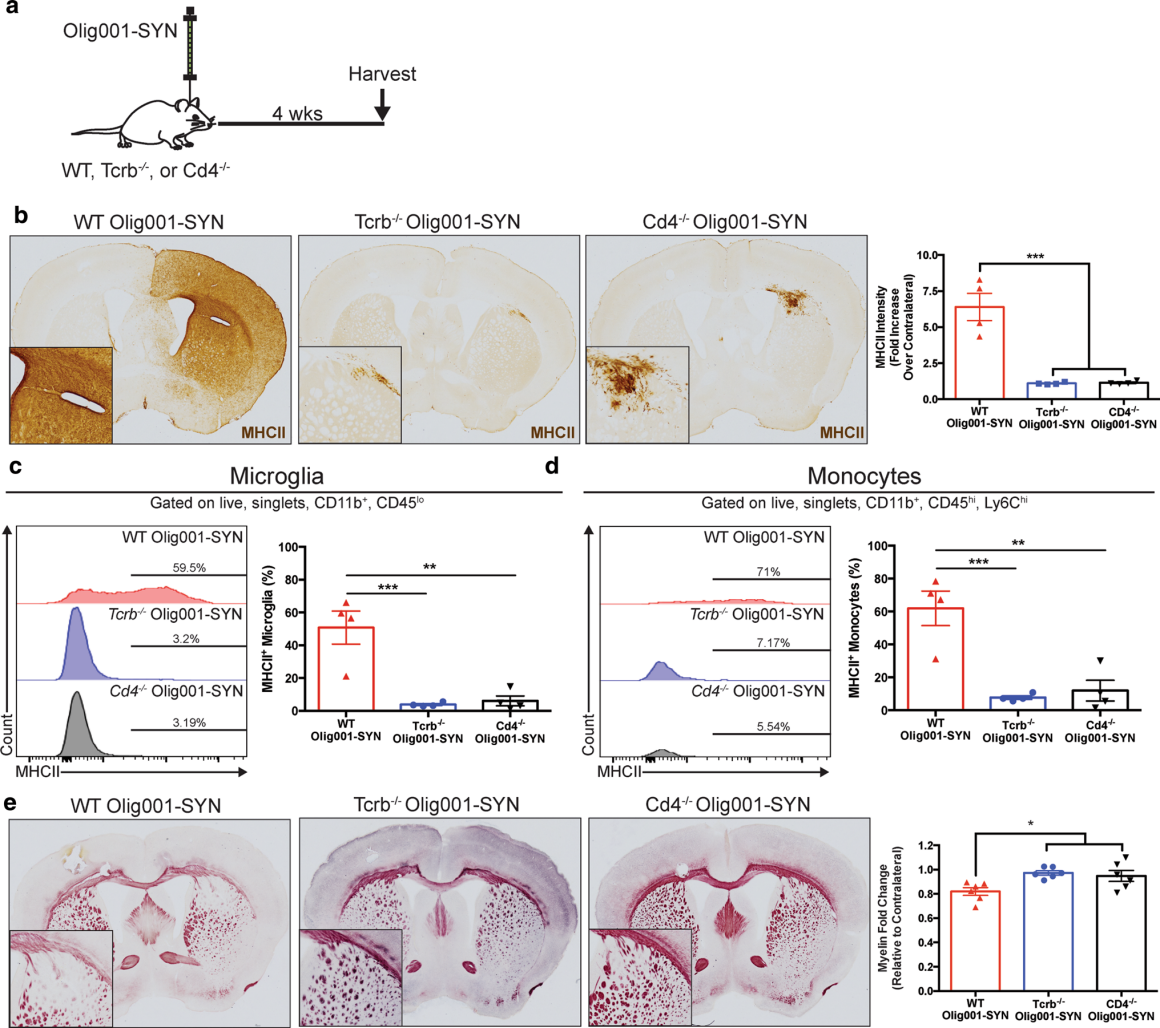
Knockout of αβ T Cells or CD4 T Cells alone reduces CNS myeloid activation and demyelination in response to α-syn expression. **a** WT, *Tcrb*^−/−^, or *Cd4*^−/−^ mice 8–12 weeks of age on a C57BL/6 J congenic background received a unilateral stereotaxic injection of Olig001-SYN into the right dorsal striatum. **b** 4 weeks post transduction MHCII^+^ immunostaining (DAB, brown) was assessed. Quantification of MHCII staining intensity relative to contralateral side in WT, *Tcrb*^−/−^, or *Cd4*^−/−^ mice transduced with Olig001-SYN. Knockout of TCRβ and CD4 attenuated Olig001-SYN induced MHCII expression. Mean values are plotted ± SEM, One way ANOVA with Dunnett’s post-hoc test ***p < 0.001, *n* = 4 per group. **c** Mononuclear isolation and flow cytometry on isolated dorsal striatal tissues from *Tcrb*^−/−^ and *Cd4*^−/−^ mice revealed a significant decrease in the percentage of CD45^lo^, CD11b^+^, MHCII^+^ microglia when compared to WT injected control. Mean values are plotted ± SEM, One-way ANOVA, Dunnett’s post-hoc test, **p < 0.01, ***p < 0.001, *n* = 4 per group. **d** Mononuclear cell isolation and flow cytometry on dorsal striatum isolated from *Tcrb*^−/−^ and *Cd4*^−/−^ revealed a significant decrease in the percent of CD45^hi^, CD11b^+^, Ly6C^hi^, MHCII^+^ monocytes when compared to WT control. Mean values are plotted ± SEM, One-way ANOVA Dunnett’s post-hoc test, **p < 0.01, ***p < 0.001, *n* = 4 per group. **e** WT, *Tcrb*^−/−^, or *Cd4*^−/−^ mice 8–12 weeks of age received a unilateral stereotaxic injection of Olig001-SYN into the right dorsal striatum. 4 weeks post transduction BlackGold staining in virally transduced mice revealed areas of active demyelination (zoom, square insert) that were attenuated in both *Tcrb*^−/−^ and *Cd4*^−/−^ mice. Mean values are plotted ± SEM, One-way ANOVA with Dunnett’s post-hoc test, *p < 0.05 *n* = 6 per group

Pro-inflammatory T cell responses contribute to the myeloid activation and tissue damage in multiple models of disease including inflammatory bowel disease [40] and multiple sclerosis [38]; and accordingly, the inflammation and pathophysiology in these models are ameliorated following knock out of these key inflammatory mediators. Given that we can observe pro-inflammatory T cell responses in our mouse model, we tested the hypothesis that knocking out T cells, or CD4 T cells more specifically, would be therapeutic for the inflammatory state and subsequent demyelination observed in our mouse model of MSA. To test this, we injected Olig001-SYN into the dorsal striatum of either WT, *Tcrb*^−/−^ (CD4 and CD8 T cell knockout), or *Cd4*^−/−^ (CD4 T cell knockout) mice and measured the CNS myeloid response via IHC and flow cytometry 4 weeks post transduction (Fig. 7a). While WT Olig001-SYN transduced mice responded with robust MHCII staining in the dorsal striatum and corpus callosum (Figs. 4, 7) *Tcrb*^−/−^ and *Cd4*^−/−^ Olig001-SYN treated mice displayed a 5.8-fold or 5.6-fold decreased (WT: 6.39 ± 0.94, *Tcrb*^−/−^: 1.1 ± 0.03, *Cd4*^−/−^: 1.14 ± 0.05, *p* = 0.0001, 0.0002) MHCII expression respectively in comparison to WT (Fig. 7b). Further, flow cytometric analysis revealed that the dorsal striatum and corpus callosum tissues in Olig001-SYN transduced *Tcrb*^−/−^ and *Cd4*^−/−^ mice displayed reduced myeloid activation as MHCII expression was significantly lower by ~ eightfold or 13-fold in microglia (WT: 50.78 ± 10.08, *Tcrb*^−/−^: 3.86 ± 0.61, *Cd4*^−/−^ 6.12 ± 3.01, *p* = 0.0008, 0.0011) (Fig. 7c) and ~ eightfold or tenfold lower in monocytes (WT: 61.95 ± 10.51, *Tcrb*^−/−^: 7.7 ± 1.1, *Cd4*^−/−^:11.93 ± 6.3, *p* = 0.0008, 0.0014) (Fig. 7d) compared to WT control. Next, to determine whether the knockout of T cells was also associated with less demyelination in response to Olig001-SYN, we performed Black Gold myelin staining similar to Fig. 3. While Olig001-SYN treated WT mice recapitulated an overall loss of myelin in the ipsilateral dorsal striatum and corpus callosum, *Tcrb*^−/−^ and *Cd4*^−/−^ treated mice possessed significantly higher myelin expression comparatively (WT: 0.81 ± 0.03, *Tcrb*^−/−^: 0.97 ± 0.01, *Cd4*^−/−^: 0.94 ± 0.04, *p* = 0.01, 0.03) (Fig. 7e). As an additional control, we measured the amount of overall pSer129 expression as well as pSer129 + cell bodies in Olig001-SYN treated WT, *Tcrb*^*−‘−*^, and *Cd4*^*−/−*^ mice and found no difference among the groups—suggesting that the genetic T cell knockouts did not have an effect on abnormal α-syn accumulation following Olig001-SYN transduction (Supplementary Fig. 9, online resource). Taken together, these data support the notion that T cells, more specifically CD4 T cells, are required for mediating the α-syn-induced inflammation and disease-driving demyelination in the dorsal striatum and corpus callosum of mice.

## Discussion

In this study, we reported the presence of CD3^+^, CD4^+^, and CD8^+^ T cells alongside the inflammatory microgliosis (HLA-DR^+^) observed in primary sites of glial cytoplasmic α-syn inclusions in the putamen and substantia nigra in MSA postmortem tissue. Additionally, we described a pro-inflammatory and disease-driving role of these T cells in a novel Olig001-SYN mouse model of MSA. Utilizing a combination of IHC and flow cytometry in an oligodendrocyte targeted α-syn expressing model of MSA we were able to complement our postmortem tissue findings and specifically implicate a role for Th1 CD4 T cells in the promotion of CNS myeloid activation and demyelination. The T cells we identified in our human postmortem tissue were of both CD4 and CD8 origin, similar to what can be observed in PD [6, 51] and more classically, demyelinating disorders such as MS [38]. These findings fit in with the body of work in neurodegenerative disease research showing increasing appreciation of the role of CNS myeloid populations (microglia and monocyte derived macrophages) and CNS lymphoid populations (T and B cells) may have in several neurodegenerative diseases including Alzheimer’s disease [27, 30, 56], PD [6, 14, 51], and amyotrophic lateral sclerosis [16, 19, 23].

Previous studies have reported positive staining for CD3^+^ T cells in the prefrontal cortex of postmortem MSA tissue as well as age matched controls [47]. Given this and the emerging role of T cells in other synucleinopathies, we sought to confirm and expand upon those findings. As previously mentioned, the area assayed in that original publication was the dorsomedial prefrontal cortex, a region thought to be more affected in the later stages of the progression of MSA [8, 60]. Looking at a more primary, early affected brain region in the putamen and substantia nigra, we found not only intense activation (HLA-DR^+^) of CNS myeloid cells (Fig. 1), but also CD3^+^ T cells, and more specifically, CD4^+^ and CD8^+^ T cells (Fig. 2a–c). It is interesting to note that the observed sum of CD4^+^ and CD8^+^ T cells does not account for all of the observed CD3^+^ T cells in MSA postmortem tissue. These CD4^−^, CD8^−^, CD3^+^ T cells could potentially be of NKT (natural killer T cell) or γδ T cell lineage and exploring their identity and function should be a focus of studies moving forward. While control subjects mainly had positive T cell staining in the blood vessels and to a lesser extent the brain parenchyma, MSA subjects showed a much greater infiltration of T cells within the brain parenchyma. Confocal microscopy revealed a close association between both HLA-DR + microglia and CD3 + T cells with pS129 + GCIs in the putamen of MSA subjects (Supplemental Figs. 1b, 3, online resource). In nigral sections, CD3^+^, CD4^+^ and CD8^+^ T cells were observed in close proximity to neuromelanin^+^ dopamine neurons, similar to observations in PD [6]. Moreover, activated microglia, as shown by HLA-DR^+^ staining and morphological differences, were seen in greater numbers near neuromelanin^+^ dopamine neurons in MSA cases compared to control subjects, as well as DARPP-32 + neurons in the putamen (Supplemental Fig. 1c). A more extensive analysis of different affected MSA-P and MSA-C brain regions is required to not only validate our findings but also to explore whether T cell infiltration is limited to only certain affected regions, or if T cell prevalence correlates with disease duration. Additionally, future studies utilizing co-staining methods could better inform interactions between microglia, macrophages, T cells, neurons, and GCIs in MSA postmortem tissue.

We extended our findings of activated myeloid cells and infiltration of CD4 and CD8 T cells into the CNS of MSA postmortem brain tissue by utilizing a novel viral model of MSA previously characterized in rodents and non-human primates [37]. Early animal models of MSA utilizing the neurotoxins 6-hydroxydopamine and quinolinic acid to help study the effects associated with the overt loss of both dopaminergic and GABAergic neurons [54, 62] found a marked induction of microglial and astrocyte activation, similar to what is seen in human MSA [29, 50]. However, with the development and adoption of α-syn based transgenic models of MSA comes the caveat that some of them do not display the hallmark of significant neuroinflammation and demyelination associated with human disease [54]. The human α-syn expressing mice under the proteolipid protein (PLP) promoter as well as A53T mutant expressing TgM83 mice inoculated with MSA patient brain homogenate do in fact display marked microglial activation [53, 60]. Similarly, the Olig001-SYN mouse model used in this study displays robust microglial activation (MHCII^+^, TMEM119^+^, CD45^lo^, CD11b^+^), but also an increased percentage of activated or inflammatory monocytes (MHCII^+^, CCR2^+^, LY6C^hi^, CD45^hi^, CD11b^+^) entering the CNS (Fig. 4). Distinguishing between the resident myeloid population and peripherally derived myeloid population during inflammatory diseases can be difficult due to the shared overlap of markers [44], but nevertheless the distinction is crucial to understanding the disease process as well as potential therapeutic targets. One limiting factor in this study is understanding the role of CNS resident microglia in disease pathogenesis in human disease and animal models of MSA. Future studies are warranted for a more descriptive analysis of CNS myeloid cells particularly, microglia, monocytes, and macrophages. Our data indicates that both populations of CNS myeloid cells (resident and peripherally derived) are inflamed, and perhaps the targeting and modulation of either population could be therapeutic in this preclinical model of MSA.

While some models of MSA display robust microglia activation, it is important to note that until this current study, no MSA model has been associated with CNS T cell infiltration. It has been observed in the neurotoxic 1-methyl-4-phenyl-1,2,3,6-tetrahydropyridine (MPTP) model of PD, that CD4^+^ T cells enter the substantia nigra and blocking or modulating that T cell response is neuroprotective [6, 46]. Similar to data acquired in PD models, our data shows that the targeted oligodendrocyte expression of α-syn results in the infiltration of both CD4 and CD8 T cells into the striatum (Fig. 5), similar to what we observed in MSA patient brain tissue (Fig. 2). It is important to note the higher proportion of CD4 T cells entering the CNS compared to CD8 T cells in response to Olig001-SYN. Indeed, we were only able to observe a trend in the increased presence of CD8 T cells, whereas CD4 T cell entry was more robust and significant. Future studies could look at later time points to determine if the CNS CD8 T cell compartment expands, similar to what was observed in MSA postmortem tissue. Nevertheless, using *Tcrb*^*−/−*^ and *Cd4*^*−/−*^ mice, we expanded our findings to show that these infiltrating T cells, and more specifically Tbet^+^, IFN-γ producing Th1 CD4 T cells (Fig. 7), are involved in mediating not only the CNS myeloid response to Olig001 SYN (Fig. 4), but also the demyelination associated with this model of MSA (Fig. 7). This pro-inflammatory, innate immune modulating, and tissue damaging role of Th1 T cells observed in our model is consistent with observations in other CNS injuries and diseases such as spinal cord injury [22] or multiple sclerosis [12].

**Fig. 8:**
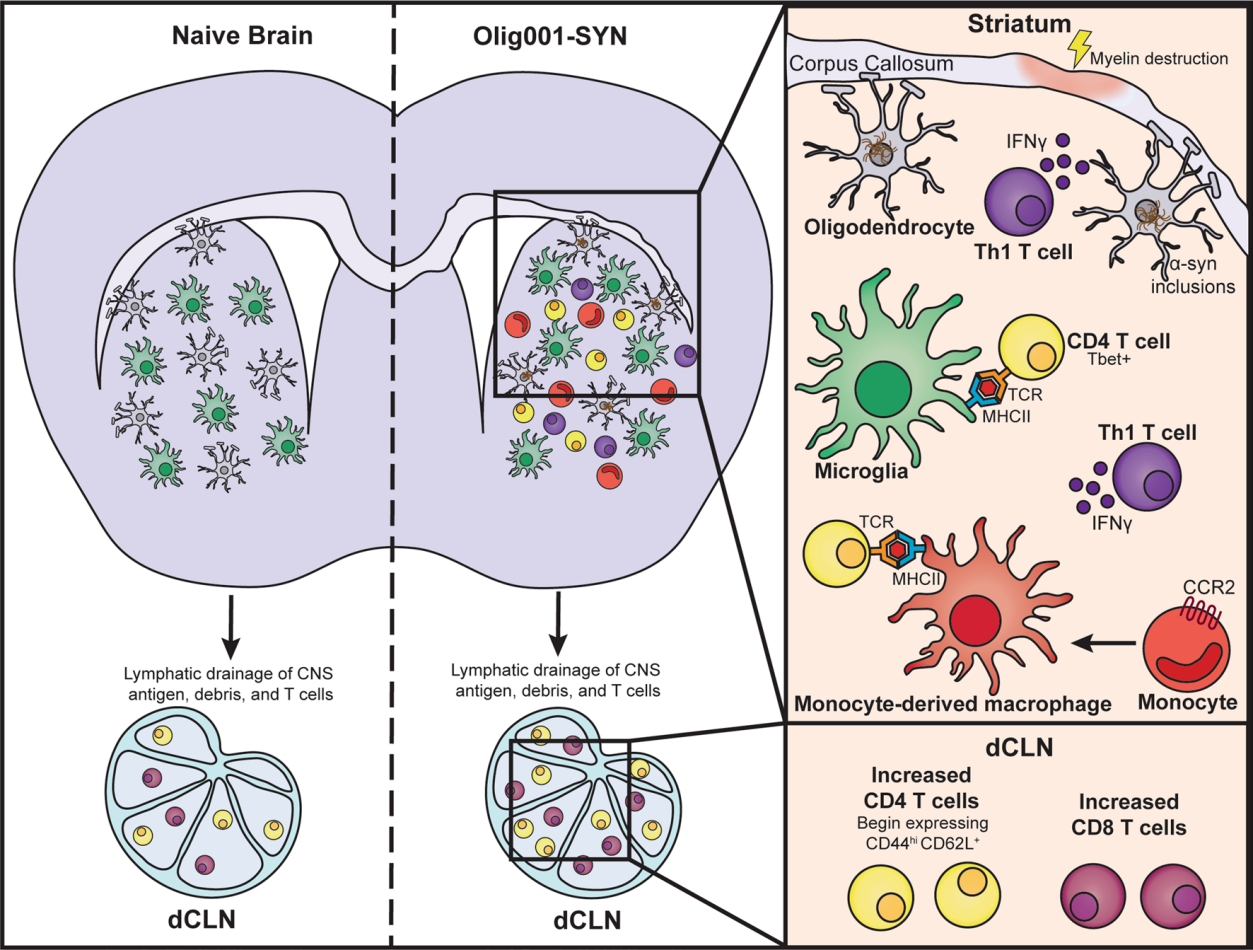
Proposed hypothesis of inflammation and neuropathology in the Olig001-SYN mouse model of MSA. The accumulation of pathogenic α-syn in oligodendrocytes (gray cells) leads to upregulation of MHCII on microglia (green cells). These activated microglia then produce signals that promote the infiltration of CD4^+^ T cells (yellow cells) and CCR2^+^ monocytes (moncyte derived macrophages, red cells) from the peripheral blood. The infiltrated CD4 T cells, now in the CNS, can interact with microglial/macrophage MHCII via their TCR. After this MHCII/TCR interaction, CD4 T cells further differentiate into Tbet^+^ Th1 T cells (purple cells) that produce IFN-γ. These CD4 T cells also drain to CNS draining deep cervical lymph nodes (dCLN) where they can potentiate further immunological responses. All of these pro-inflammatory cells (microglia, monocyte-derived macrophages, and CD4 T cells) promote dysfunction/destruction of oligodendrocytes leading to demyelination of the striatum and corpus callosum

One hypothesis (Fig. 8) connecting some of our findings is that oligodendrocyte mediated expression of α-syn causes not only oligodendrocyte dysfunction [13, 17, 58], but also local microglial activation. Both microglia and oligodendroglia are capable of emitting pro-inflammatory danger signals that can further damage surrounding oligodendrocyte precursor cells (OPCs) and neurons. CNS antigens from the cellular debris produced by these damaged OPCs, neurons, or oligodendroglia are drained to the cervical lymph node via the meningeal lymphatics [11, 35]. Once in the draining lymph node, an antigen presenting cell loads a CNS antigen, possibly α-syn, onto the MHCII which is misrecognized by an auto-antigenic CD4 T cell. This Olig001-SYN associated CD4 T cell then clonally expands and exits the draining lymph node and follows chemokine signals back into the injured CNS. Now in the CNS, these Olig001-SYN associated CD4 T cells are reactivated by an MHCII^+^ microglia displaying the same CNS antigen. This interaction induces a signal cascade that includes the secretion of IFN-γ which can primarily serve to further push microglia to a pro-inflammatory phagocytic state [41], support the chemotaxis of peripheral inflammatory monocytes to the CNS [15], and cause damage to surrounding neurons or OPCs [31, 32, 41] (Fig. 8). Accordingly, our experiments using T cell knockout mice demonstrated the integral role of T cells to this neurotoxic response to oligodendrocyte expression of α-syn. Substantial studies are required to support this hypothesis and include identifying the chemokine signals being produced by dysfunctional oligodendroglia, the role of astrocytes in this inflammatory cascade, and the identity of the CNS target of these CD4 T cells. Further studies are also required to determine the effect of α-syn overexpression on the differentiation and myelinating capacity of oligodendrocytes/OPCs. Additionally, as no noticeable cell loss occurred in the 4-week time point utilized in these studies, future studies that prioritize longer time points will be necessary to determine if synaptic degeneration, neuronal cell loss, and behavioral deficits exist in the Olig001 model.

Altogether, our data show infiltrating CD4 and CD8 T cells alongside HLA-DR^+^ microglia in MSA-C and MSA-P postmortem brain tissue. We corroborated these findings using a novel mouse model of MSA which selectively overexpresses α-syn in oligodendrocytes via an Olig001 vector system in the dorsal striatum. The model was able to recapitulate the activated myeloid (microglia and monocytes) system as well as the adaptive (CD4 and CD8 T cells) response observed in postmortem samples. Furthermore, we described a key role of Th1 CD4 T cells in modulating the neuroinflammatory myeloid and demyelinating response to Olig001-SYN. These data better inform potential immunotherapeutics in the Olig001-SYN model as well as other preclinical models of MSA that display hallmark inflammation. Broadly, this study provides a new rationale for the design and testing of clinical immunotherapies targeting potentially pro-inflammatory T cells in patients with MSA, an aggressively debilitating and fatal disease with no impactful treatments.

## Electronic supplementary material

Below is the link to the electronic supplementary material.

Supplementary file1 Table displaying the subject demographics of the postmortem tissue samples used for the HLA-DR, CD3, CD4, and CD8 immunohistochemical stainings (DOCX 15 kb)

Supplementary file2 (DOCX 89160 kb)
