# Supplementary material for: T cell infiltration in both human multiple system atrophy and a novel mouse model of the disease
Source: Acta Neuropathol. 2020 Jan 29;139(5):855–74. doi: 10.1007/s00401-020-02126-w (PMC7181566; doi:10.1007/s00401-020-02126-w)
Supplement: Supplementary file 1 — Supplementary file1 Table displaying the subject demographics of the postmortem tissue samples used for the HLA-DR, CD3, CD4, and CD8 immunohistochemical stainings (DOCX 15 kb) [file 401_2020_2126_MOESM1_ESM.docx]

Table related to *Acta Neuropathologica* manuscript:

**T cell infiltration in both human multiple system atrophy and a novel mouse model of the disease**

Gregory P. Williams^1†^, David J. Marmion^2†^, Aubrey M. Schonhoff^1^, Asta Jurkuvenaite^1^, Woong-Jai Won^1^, David G. Standaert^1^, Jeffrey H. Kordower^2^, Ashley S. Harms^1^*

^1^:Center for Neurodegeneration and Experimental Therapeutics, Department of Neurology, The University of Alabama at Birmingham, Birmingham, AL, USA, 35294. ^2^: Department of Neurological Sciences, Rush University Medical Center, Chicago, IL, USA, 60612. *Corresponding Author: Ashley S. Harms, PhD Assistant Professor of Neurology, Center for Neurodegeneration and Experimental Therapeutics University of Alabama at Birmingham (UAB), 1719 6th Ave. South, CIRC 446 Birmingham, AL 35294-0021 Phone 205-934-6142 Fax 205-996-6580. ^†^These authors contributed equally: Gregory P. Williams, David J. Marmion

| **Table 1. Subject Demographics** | | | | | |
| --- | --- | --- | --- | --- | --- |
| **Case ID** | **Age** | **Sex** | **PMI (hrs)** | **Diagnosis** | **GCI** |
| B13-053 | 56 | M | 8.3 | MSA^2^ | Yes |
| B15-010 | 75 | F | 3 | MSA^3^ | Yes |
| B11-004 | 64 | M | 4.1 | MSA^4^ | Yes |
| 07-36 | 75 | M | 2.83 | MSA^5^ | Yes |
| 04-56 | 74 | M | 3.66 | MSA^5^ | Yes |
| 04-51 | 64 | F | 2.16 | MSA^5^ | Yes |
| 01-44 | 78 | F | 2.83 | Neurological Control^1^ | No |
| 02-04 | 80 | F | 2 | Neurological Control^1^ | No |
| 02-33 | 85 | M | 2.25 | Neurological Control^1^ | No |
| 09-57 | 80 | M | 3.5 | Neurological Control^1^ | No |
| 98-37 | 78 | M | 2.25 | Neurological Control^1^ | No |
| 18-78 | 71 | M | 3.5 | Neurological Control^1^ | No |
| ^1^Neurological controls are subjects that did not present with dementia or parkinsonism during life and had no major neuropathological diagnosis after neuropathology evaluation postmortem.  ^2^ Diagnosed with MSA-P  ^3^ Diagnosed with mixed MSA (Parkinsonian motor features)  ^4^ Diagnosed with MSA-C  ^5^ Neuropathological exam confirmed MSA, no information available on subtype | | | | | |
