# Supplementary material for: T cell infiltration in both human multiple system atrophy and a novel mouse model of the disease
Source: Acta Neuropathol. 2020 Jan 29;139(5):855–74. doi: 10.1007/s00401-020-02126-w (PMC7181566; doi:10.1007/s00401-020-02126-w)
Supplement: Supplementary file 2 — Supplementary file2 (DOCX 89160 kb) [file 401_2020_2126_MOESM2_ESM.docx]

Supplementary material for *Acta Neuropathologica* manuscript:

**T cell infiltration in both human multiple system atrophy and a novel mouse model of the disease**

Gregory P. Williams^1†^, David J. Marmion^2†^, Aubrey M. Schonhoff^1^, Asta Jurkuvenaite^1^, Woong-Jai Won^1^, David G. Standaert^1^, Jeffrey H. Kordower^2^, Ashley S. Harms^1^*

^1^:Center for Neurodegeneration and Experimental Therapeutics, Department of Neurology, The University of Alabama at Birmingham, Birmingham, AL, USA, 35294. ^2^: Department of Neurological Sciences, Rush University Medical Center, Chicago, IL, USA, 60612. *Corresponding Author: Ashley S. Harms, PhD Assistant Professor of Neurology, Center for Neurodegeneration and Experimental Therapeutics University of Alabama at Birmingham (UAB), 1719 6th Ave. South, CIRC 446 Birmingham, AL 35294-0021 Phone 205-934-6142 Fax 205-996-6580. ^†^These authors contributed equally: Gregory P. Williams, David J. Marmion


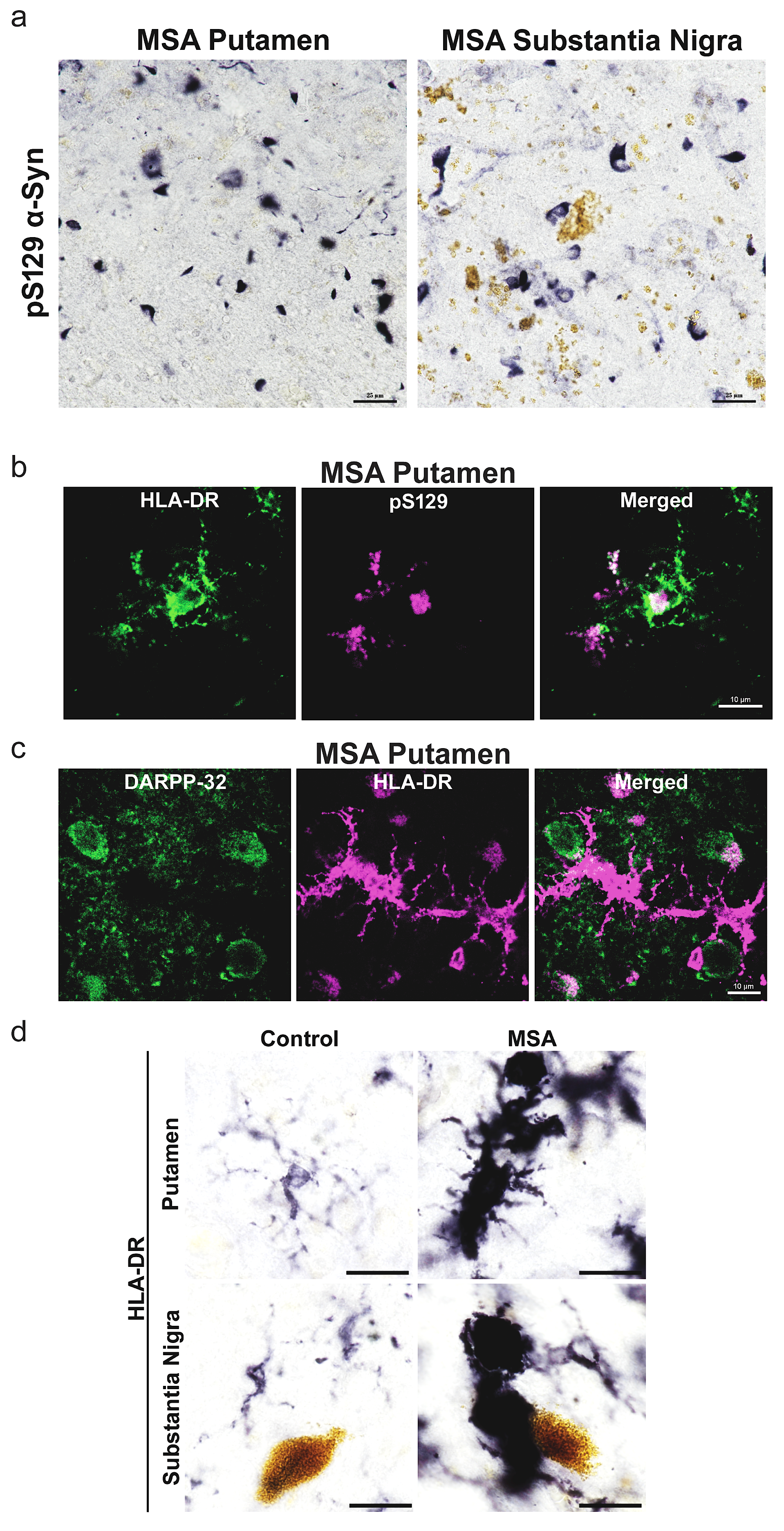


**Supplementary Fig. 1:**

(a) Representative images confirming the presence of pSer129+ alpha-synuclein GCIs (DAB, black) in the putamen (left panel) and substantia nigra (right panel) of MSA postmortem tissue. GCIs are not present in Control cases (data not shown). Scale bar is 25 μm. (b) Immunofluorescent images confirming HLA-DR reactive microglia (green) surrounding pSer129+ GCI (purple) in the putamen of MSA post-mortem tissue. Scale bar is 10 μm. (c) Immunofluorescent images confirming the presence of HLA-DR reactive microglia (purple) in the presence of DARP-32+ neurons (green) in the putamen of MSA post mortem tissue. Scale bar is 10 μm. (d) High magnification images displaying enhanced HLA-DR reactivity (DAB, black) and morphology compared to neurological control in the MSA post mortem putamen (top panel) and substantia nigra (bottom panel). Brown pigmentation are neuromelanin laden dopamine neurons in the substantia nigra. Scale bar is 20 μm. Representative images.


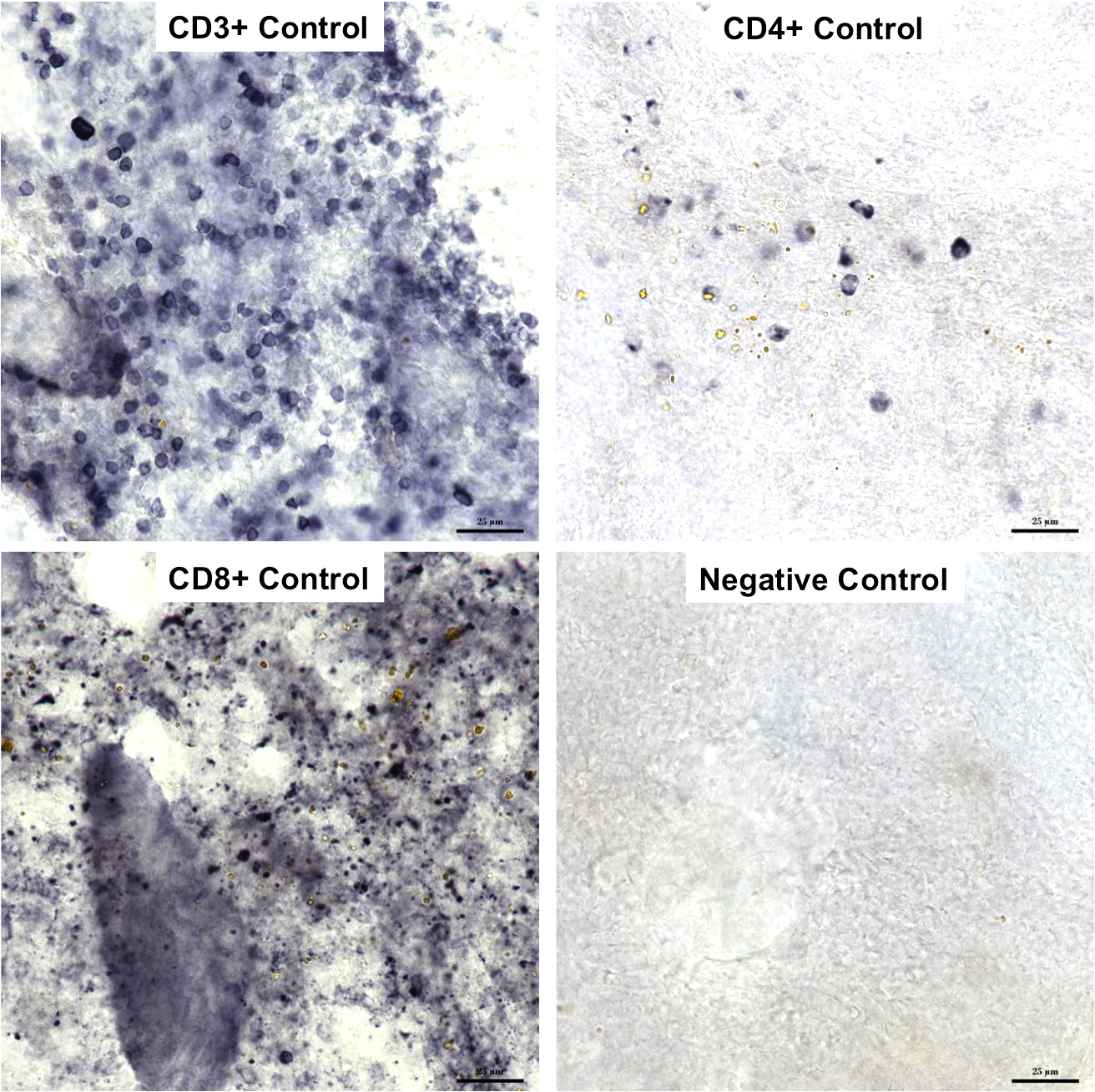


**Supplementary Fig. 2**

Cynomolgus macaque spleen was used as a positive control for CD3, CD4, and CD8 immunohistochemical staining. Negative control indicates tissue sections that underwent entire staining protocol except primary antibody incubation. Scale bars are 25 μm. Representative images.


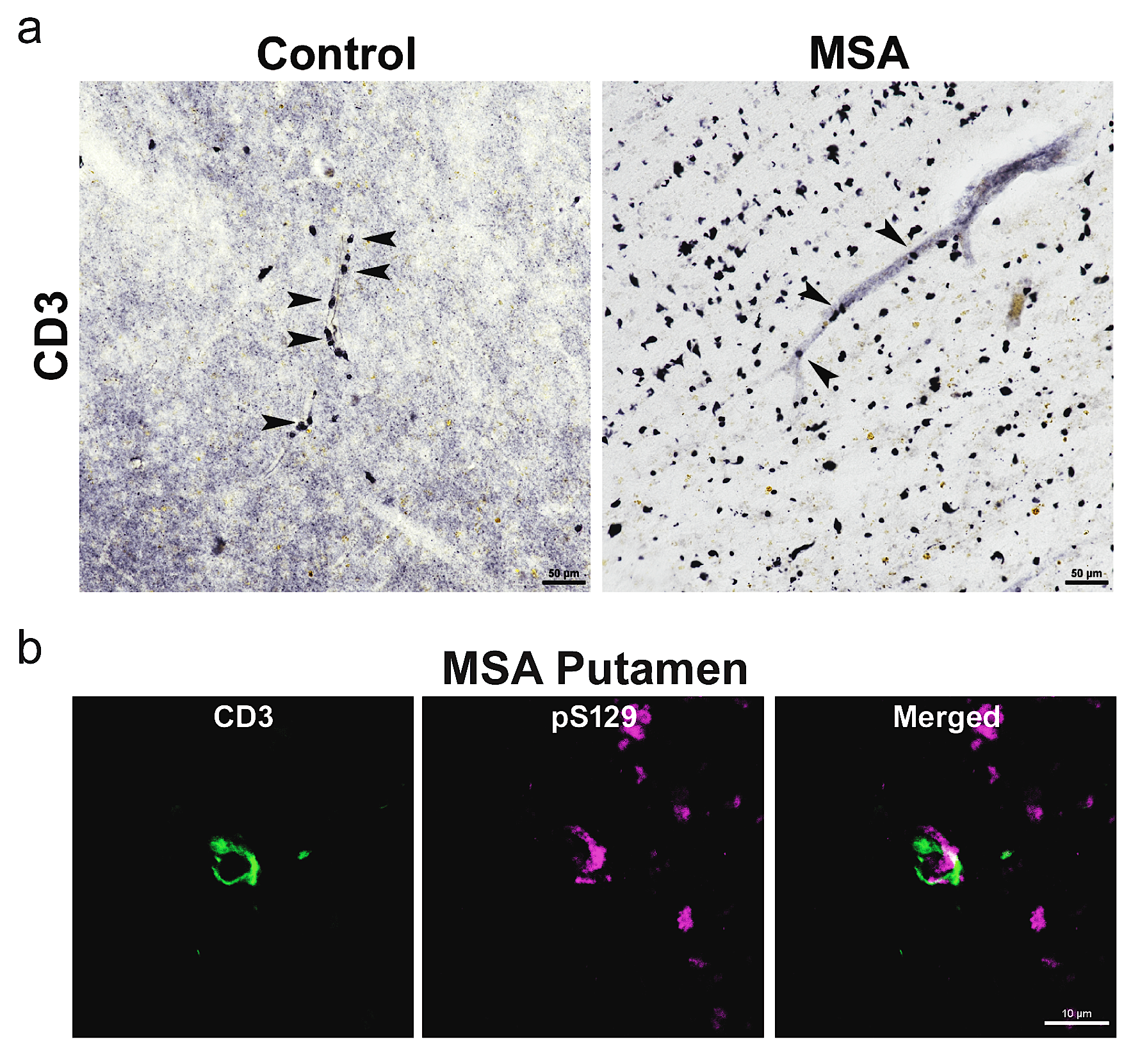


**Supplementary Fig. 3**

1. CD3 IHC (DAB, black) detailing the presence of CD3+ T cells primarily surrounding areas of the vasculature (black arrow heads) in the post-mortem putamen of neurological controls (left panel). CD3+ T cells are present in areas of the vasculature (black arrow heads) and much more abundant within the brain parenchyma in the putamen of MSA post mortem tissue (right panel). Representative images, scale bar is 50 μm. (b) Immunofluorescent images confirming CD3 reactive T cells (green) surrounding pSer129+ GCI (purple) in the putamen of MSA post-mortem tissue. Scale bar is 10 μm.


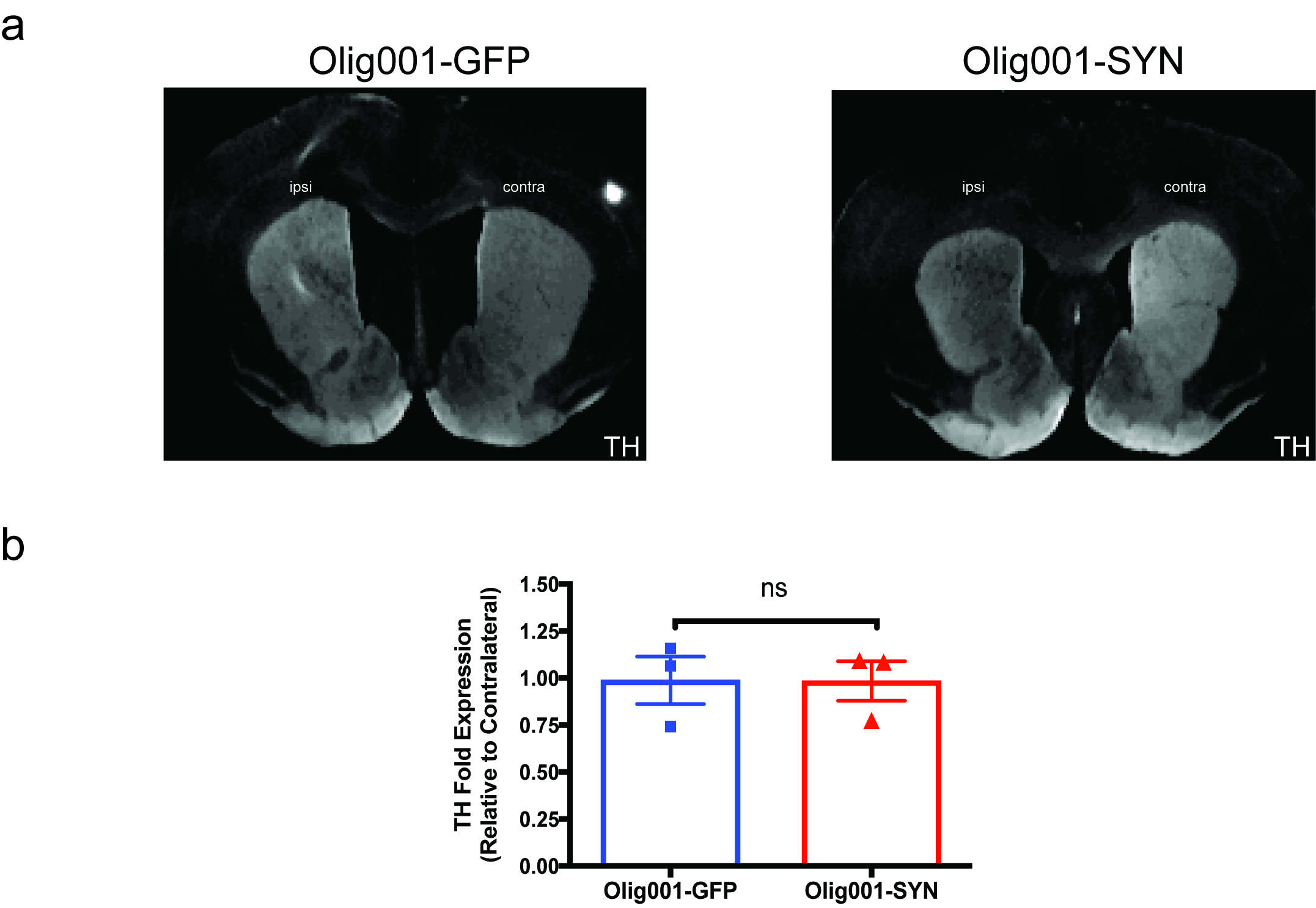


**Supplementary Fig. 4**

C57BL/6J mice 8-12 weeks of age received a unilateral stereotaxic injection of Olig001-SYN or Olig001-GFP control into the dorsal striatum. 4 weeks post-transduction (a) Representative striatal sections from Olig001-GFP (left panel) and Olig001-SYN (right panel) injected mice immunolabeled with TH (white). (b) densitometry analysis for TH expression in the ipsilateral (ipsi) striatum. Mean values are plotted relative to contralateral ± SEM. Unpaired t-test. n=3 per group ns=not significant.


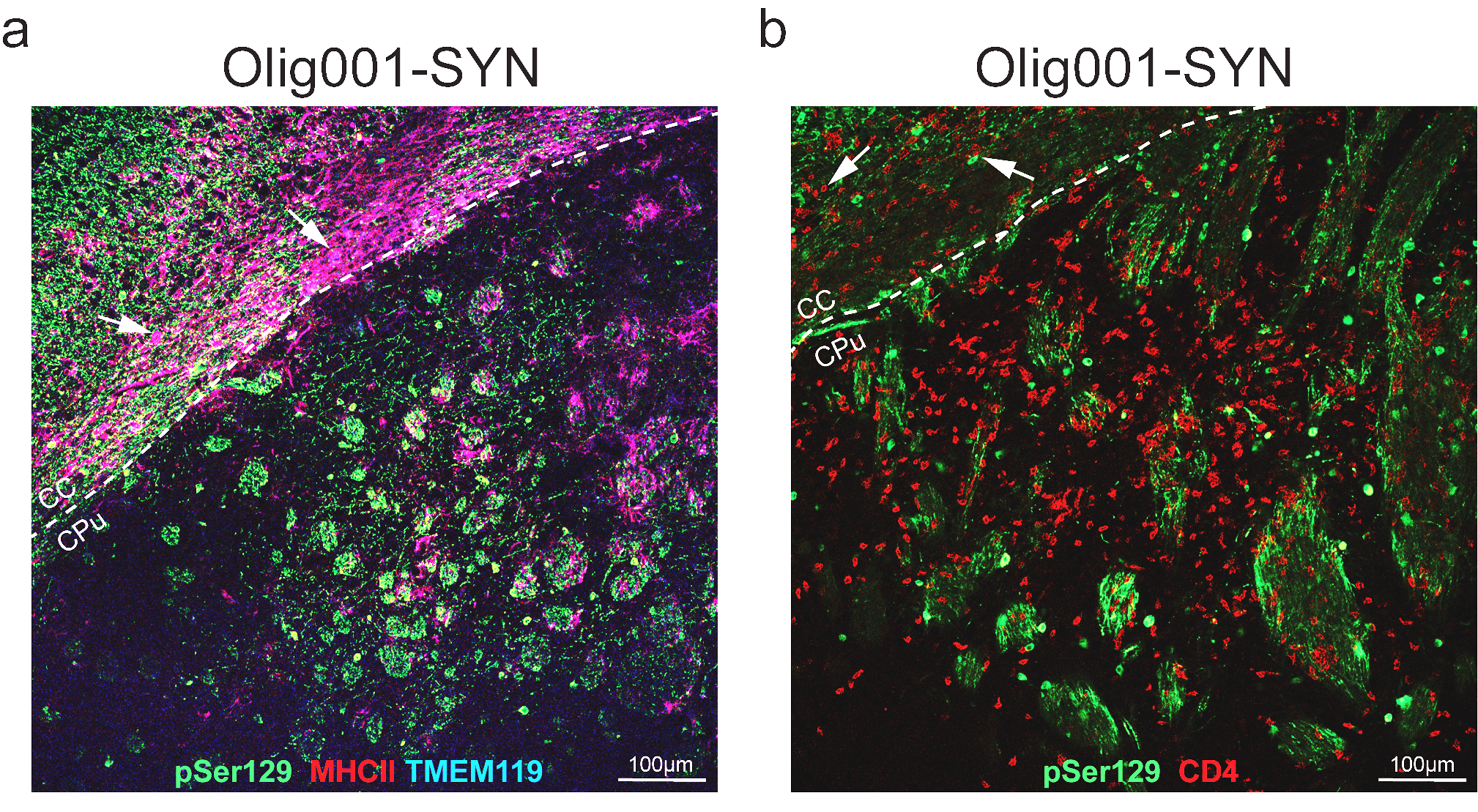


**Supplementary Fig. 5**

C57BL/6J mice 8-12 weeks of age received a unilateral stereotaxic injection of Olig001-SYN into the dorsal striatum. 4 weeks post-transduction IHC highlighting the presence of (a) white arrows display activated microglia (TMEM119+, blue; MHCII+, red) and (b) T cells (CD4+, red) in both the striatum (labeled CPu) and corpus callosum (CC). Representative images. Scale bar is 100 μm.


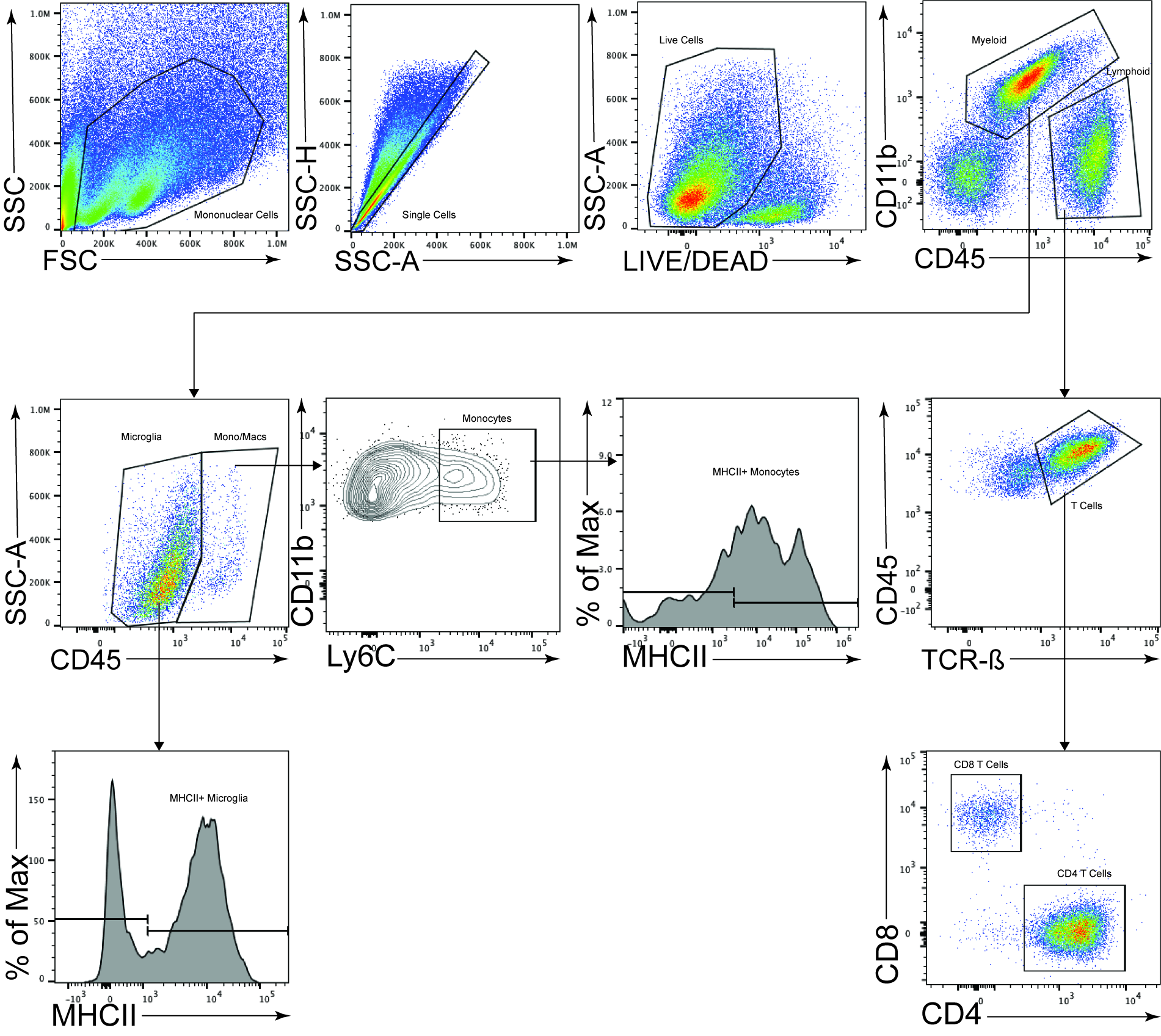


**Supplementary Fig. 6**

Gating strategy used for flow cytometric analysis on mononuclear cells isolated from the dorsal striatum and corpus callosum of Olig001 injected mice.


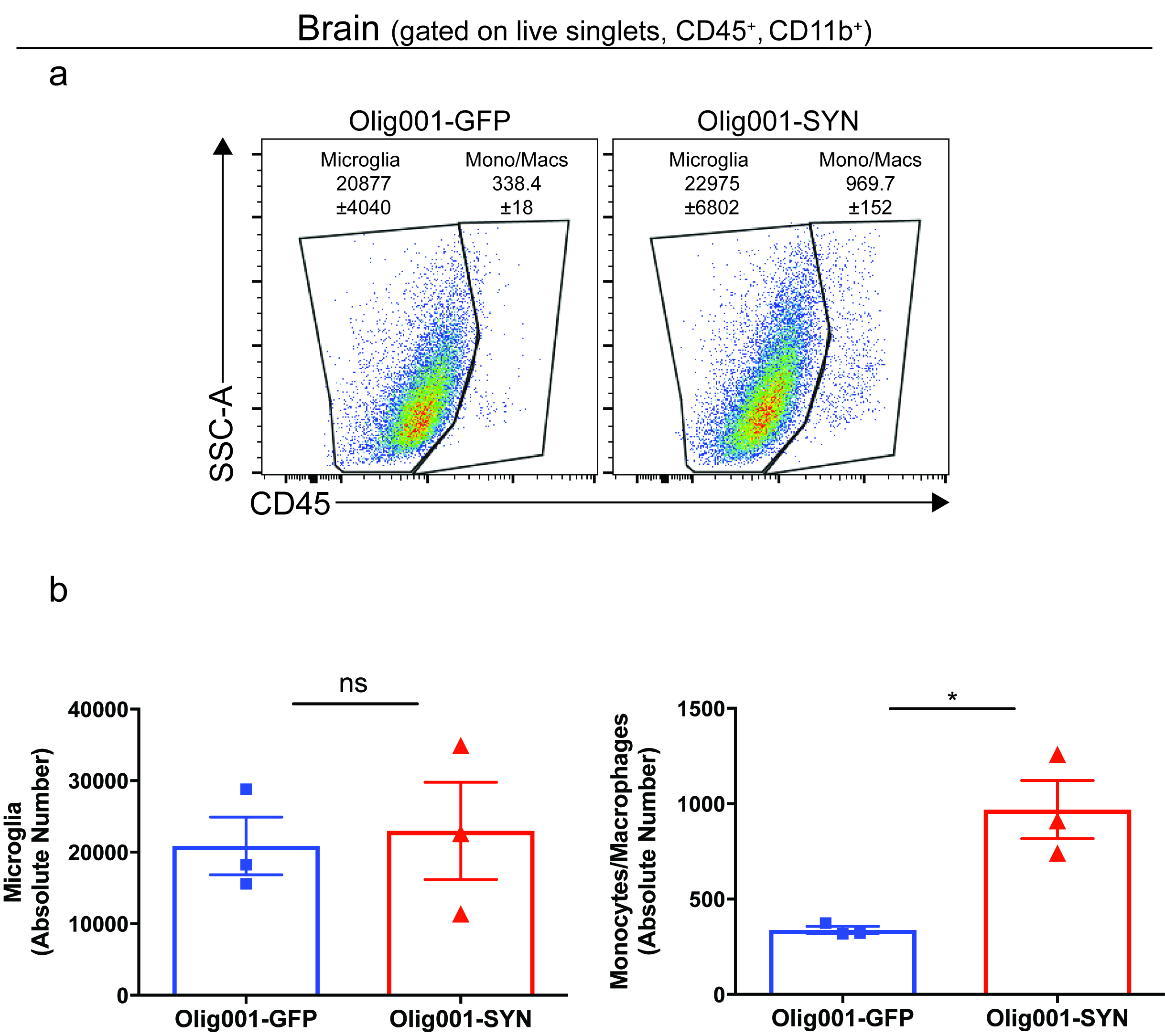


**Supplementary Fig. 7**

C57BL/6J mice 8-12 weeks of age received a bilateral stereotaxic injection of Olig001-SYN or Olig001-GFP control into the dorsal striatum. 4 weeks post-transduction in the CNS, mononuclear cell isolation, cell surface staining, and flow cytometry were performed on the isolated dorsal striatal tissues. (a) Representative flow cytometry plots depicting partial gating of the stained microglia (CD45^lo^, CD11b^+^) and monocyte/macrophage populations (CD45^lo^, CD11b^+^). (b) Quantification of the microglia and monocyte/macrophage staining in (a). Mean values are plotted + SEM, unpaired t-test, *p<0.05, n=3 per group.


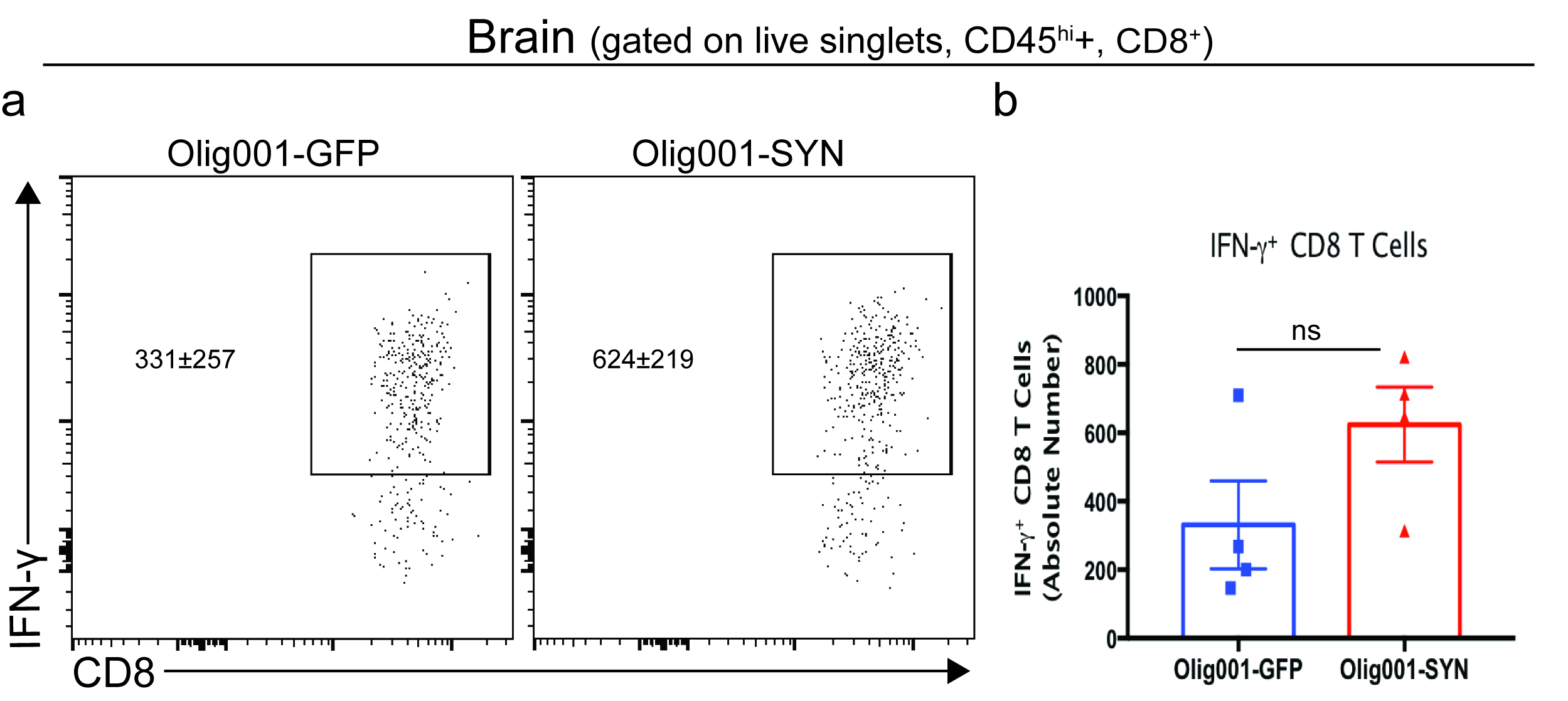


**Supplementary Fig. 8**

WT mice 8-12 weeks of age received a bilateral stereotaxic injection of Olig001-SYN or Olig001-GFP control into the dorsal striatum. 4 weeks post-transduction in the CNS, mononuclear cell isolation, intracellular cytokine, transcription factor staining, and flow cytometry were performed on the isolated dorsal striatal tissues. (a) Representative flow cytometry plots depicting positive gating of the stained cytokine IFNγ-in CD8 T cells. (b) Quantification of cytokine staining in (a). Mean values are plotted + SEM, unpaired t-test, ns=not significant, n=4 per group.


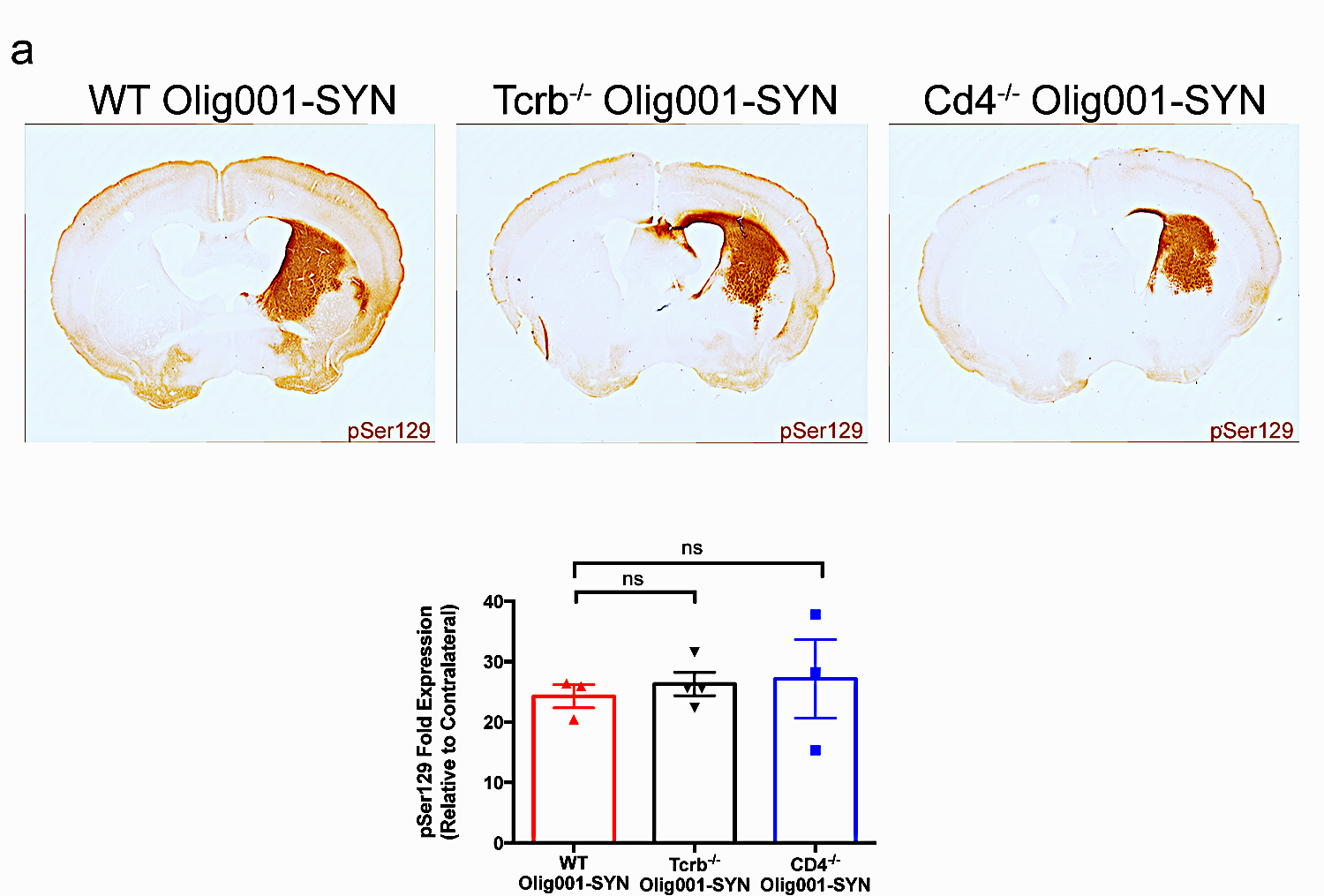


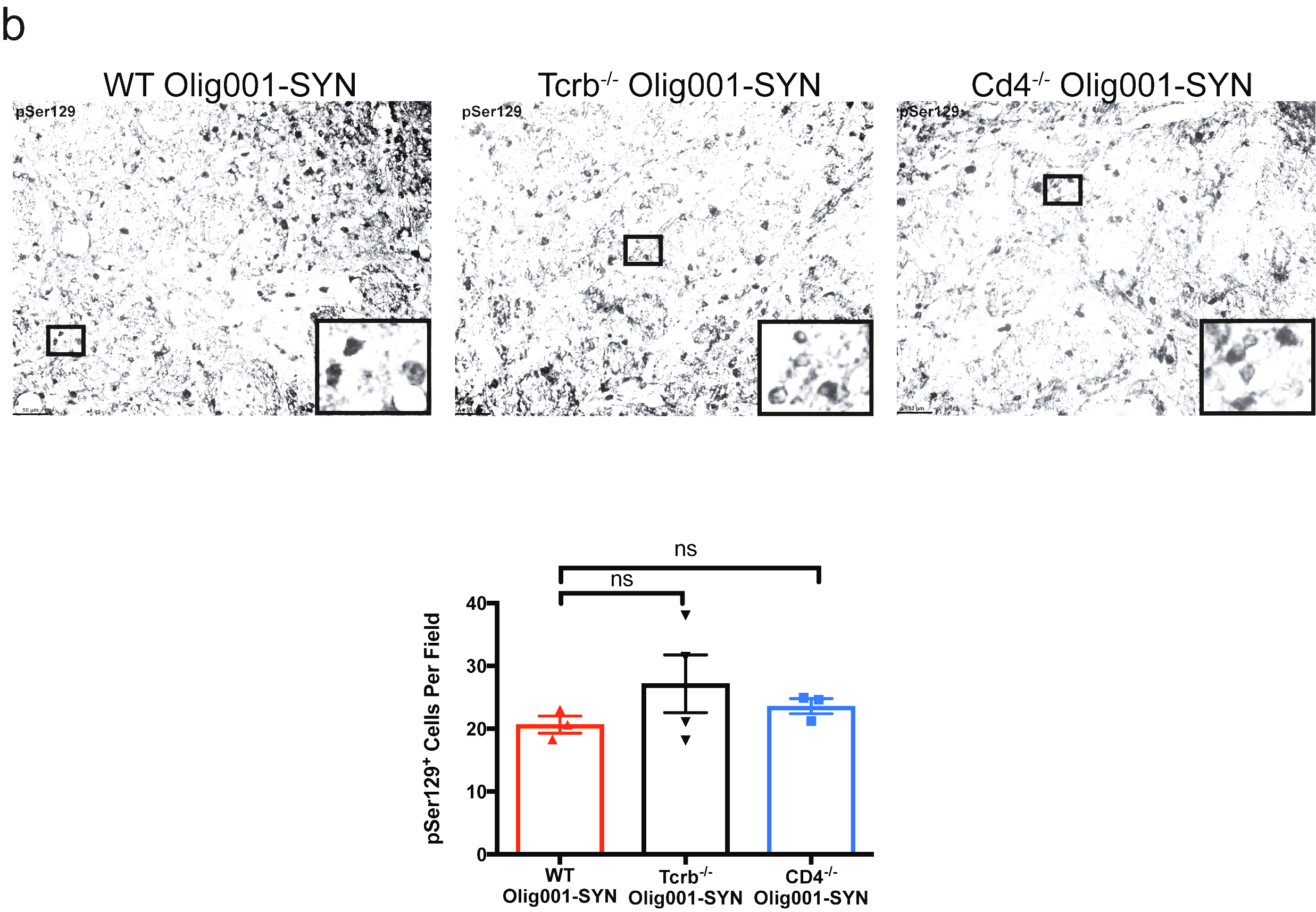


**Supplementary Fig. 9**

C57BL/6J mice 8-12 weeks of age received a unilateral stereotaxic injection of Olig001-SYN into the dorsal striatum. 4 weeks post-transduction (a) representative images and quantification of pSer129 immunohistochemical staining intensity in WT, Tcrb^-/-^, or Cd4^-/-^ mice transduced with Olig001-SYN. Knockout of TCR-β or CD4 did not affect Olig001-SYN induced pSer129 expression. Mean values are plotted ± SEM, One way ANOVA, ns=not significant, n=3-4 per group. (b) Representative images and unbiased MSAquantification of pSer129+ glial cytoplasmic inclusions in WT, Tcrb^-/-^, or Cd4^-/-^ mice transduced with Olig001-SYN. Knockout of TCR-β or CD4 did not affect the average number of Olig001-SYN induced pSer129 GCIs. Mean values are plotted ± SEM, One way ANOVA, ns=not significant, n=3-4 per group.
